# Large-scale placenta DNA methylation integrated analysis reveals fetal sex-specific differentially methylated CpG sites and regions

**DOI:** 10.1038/s41598-022-13544-z

**Published:** 2022-06-07

**Authors:** Shan V. Andrews, Irene J. Yang, Karolin Froehlich, Tomiko Oskotsky, Marina Sirota

**Affiliations:** 1 Bakar Computational Health Sciences Institute, UCSF, San Francisco, CA USA; 2 Dougherty Valley High School, San Ramon, CA USA; 3 Department of Pediatrics, UCSF, San Francisco, CA USA

**Keywords:** Computational biology and bioinformatics, Developmental biology

## Abstract

Although male–female differences in placental structure and function have been observed, little is understood about their molecular underpinnings. Here, we present a mega-analysis of 14 publicly available placenta DNA methylation (DNAm) microarray datasets to identify individual CpGs and regions associated with fetal sex. In the discovery dataset of placentas from full term pregnancies (N = 532 samples), 5212 CpGs met genome-wide significance (*p* < 1E−8) and were enriched in pathways such as keratinization (FDR *p*-value = 7.37E−14), chemokine activity (FDR *p*-value = 1.56E−2), and eosinophil migration (FDR *p*-value = 1.83E−2). Nine differentially methylated regions were identified (fwerArea < 0.1) including a region in the promoter of *ZNF300* that showed consistent differential DNAm in samples from earlier timepoints in pregnancy and appeared to be driven predominately by effects in the trophoblast cell type. We describe the largest study of fetal sex differences in placenta DNAm performed to date, revealing genes and pathways characterizing sex-specific placenta function and health outcomes later in life.

## Introduction

The placenta is a key organ during pregnancy, performing important functions such as providing nutrients, transferring respiratory gases, and secreting hormones for adequate fetal growth and development^1^. During pregnancy, the placenta grows and changes in composition and function, developing within the mother, but is primarily regulated by the fetal genome^1,2^. Epidemiologic evidence suggests that the periconception and in utero periods are the most vulnerable to environmental factors influencing the susceptibility for several diseases later in life^3,4^. The placenta, as the key mediator of the gestational environment, therefore has a significant role in the fetal programming of health outcomes into adulthood^5^. Further, observed differences by sex with respect to prevalence or severity in these health outcomes, as has been observed for autism spectrum disorder (ASD)^6^, schizophrenia^7^, and autoimmune diseases^8^, among many other examples, may be influenced by sex-specific gestational environments that are mediated by sex-specific placenta structure and function.

Some male–female differences during in utero fetal development and childbirth are already known. For example, male fetuses are characterized by higher birth weight, placenta weight, and birth weight to placenta weight ratio^9,10^. Moreover, males have a higher risk of suffering from peri- and postnatal complications^11–16^. Previous studies have demonstrated that male and female fetuses utilize different mechanisms to cope with adverse intrauterine environments^17–20^. For instance, in the presence of chronic maternal asthma growth of female fetuses is reduced, resulting in neonates of smaller size and lower birth weight compared to neonates from healthy pregnancies^18,21^. Any further complication during pregnancy, such as an acute maternal asthma exacerbation, did not decrease their survival rate, indicating a vital adaptation mechanism of female fetuses^21,22^. In contrast, growth of male fetuses appears not to be reduced in the presence of maternal chronic asthma; however, male fetuses experienced a higher rate of adverse outcomes among women who had a severe asthma exacerbation^18,20^.

A growing body of literature has sought to understand the placental molecular mechanisms at play in disease outcomes or complicated pregnancies. Placenta gene expression studies have examined differences in preeclampsia^23,24^, gestational diabetes mellitus^25^, and intrauterine growth restriction (IUGR)^26^. For DNA methylation (DNAm), examples of studies include those examining preterm birth^27^, ASD^28^, and also preeclampsia^29^ and IUGR^30^. There have been some examples in these studies of analyses that explicitly seek to quantify sex-specific mechanisms. For example, gene expression differences, including for various cytokines, have been shown to characterize fetal sex-specific responses to maternal asthma exposure^31–33^. However, these sex-focused analyses are rare, and there have been few studies that have examined sex itself as an “outcome” in analyses, even in normal or non-pathological placentas. Finally, the majority of transcriptomic and DNAm studies of the placenta have not thoroughly accounted for or investigated the role of cell type heterogeneity in phenotypic studies^34^, though a recent study by Yuan et al.^35^ has helped to address this gap by generating DNAm profiles in the four major placenta cell types. Specifically, this study profiled endothelial cells, placental macrophages known as Hofbauer cells, mesenchymal stromal cells and trophoblasts, composed of both syncytiotrophoblasts that interact with maternal blood and cytotrophoblasts that invade the uterine spiral arteries^36^. Overall, the mechanisms by which placental molecular factors act in a sex-specific manner to determine fetal phenotypes, and how these mechanisms act through specific cell types or change with gestational age, remain as critical gaps in our understanding of developmental health. While placental gene expression studies investigating sex-specific differences have been undertaken^37–40^, previous studies of placenta DNAm related to fetal sex^41,42^ have suffered from small sample size. Finally, a recent study by Inkster et al. did address previous limitations of placenta DNAm studies of fetal sex by pooling data from many publicly available datasets^43^. As this study was limited to full term placenta samples, there remains a lack of understanding of how full term placenta sex differences also exist during earlier points in gestation.

Here, we present a mega-analysis of publicly available placenta DNAm samples from uncomplicated pregnancies in order to maximize sample size for identification of sex-specific differences. Whereas meta-analyses use summary statistics as inputs, mega-analyses pool raw data from multiple studies that were conducted under comparable conditions^44^. We first sought to characterize sex-specific patterns in DNAm in full term placentas, aiming to identify differentially methylated CpG sites as well as differentially methylated regions (DMRs). We then performed similar analyses using placenta samples from earlier points in pregnancy (1st, 2nd, and 3rd trimesters individually) and evaluated which findings from full term placentas persist across the gestational period. Finally, we examined our results in light of recently published cell type specific placenta DNAm patterns, to understand the degree to which cell type heterogeneity drove our findings and if these patterns could shed light on the mechanism of our identified sites and regions of interest.

## Results

### Sex-specific methylation patterns of non-pathological human placenta tissue

**Figure 1:**
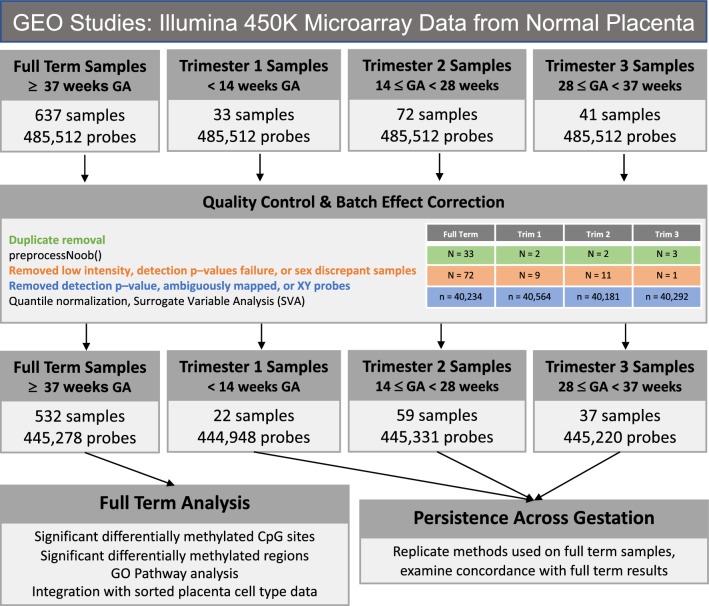
Study design and methodology. Illumina 450K datasets of fetal placenta samples were downloaded from GEO and restricted to those from normal/typical pregnancies to the fullest extent possible (see “Methods”). Samples were classified into 4 groups according to annotated gestational age, and each dataset was processed through identical subject and probe level QC steps. Single site and region-based analyses were performed in full term samples first, and then also performed in individual trimester datasets to examine replication of full term findings. See “Methods” for additional details.

We analyzed sex differences in human placenta DNAm patterns incorporating 783 samples from 14 Illumina 450K array GEO data sets (Fig. 1, Supplementary Table 1). Samples from control and/or normal pregnancies (defined to the fullest extent possible based on available metadata, see “Methods”) were binned into groups according to gestational period. The full term dataset was treated as the discovery dataset, owing to its large sample size. The smaller, less-statistically powered individual trimester datasets were treated as replication cohorts examining the extent to which differentially methylated sites and regions identified at full term persisted throughout the gestational period. In the full term dataset and specific trimester datasets, individual CpG sites and regions were evaluated for their association with fetal sex, adjusting for dataset-specific surrogate variables (see “Methods”). We verified that the surrogate variables captured batch effects resulting from the many individual studies that contributed to the datasets (Supplementary Figs. 1–4).

### Differentially methylated CpG sites by fetal sex in full term placenta samples

**Figure 2:**
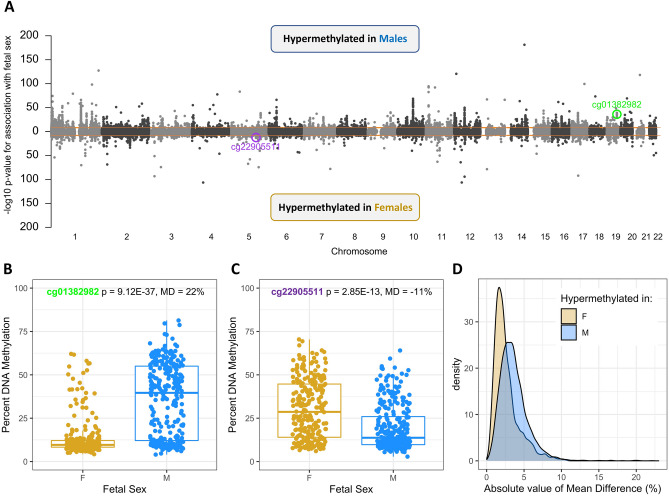
Single site discovery in full term placenta samples. (**A**) Manhattan plot representing results from full term samples. Top half restricted to sites hypermethylated in males, bottom half restricted to sites hypermethylated in females. Orange line represents threshold of 1E−8, the considered cutoff for genome-wide significance. (**B**) Boxplots of percent methylation in males (blue) and females (gold) at cg01382982, the genome-wide significant CpG site at which males were most hypermethylated relative to females (Mean Difference, MD = 22%). (**C**) Boxplots of percent methylation in males (blue) and females (gold) at cg22905511, the genome-wide significant CpG site at which females were most hypermethylated relative to males (Mean Difference, MD = − 11%). (**D**) Distribution of absolute value of mean differences in sites hypermethylated in males (blue, n = 3793) and sites hypermethylated in females (gold, n = 1419).

We did not observe global DNAm differences by fetal sex in genomic regions defined by relation to CpG islands or ENCODE-determined regulatory features (Supplementary Fig. 5A–B). Single site analysis at 445,278 autosomal in 532 full term placenta samples (after QC; see “Methods”) revealed that a total of 5212 CpG sites were significantly associated with fetal sex at a genome wide significance threshold of 1E-8 (Fig. 2A, Supplementary Fig. 6A, Supplementary Data 1). These 5212 sites tended to be enriched for probes mapping to open sea regions, depleted for probes mapping to CpG islands, and depleted for probes mapping to promoter regions compared to the full list of probes examined in the differential DNAm analysis (Supplementary Fig. 5C–D). Of these, cg01382982, harbored in the CpG island promoter of *ZNF175*, exhibited the largest degree of hypermethylation in males (Fig. 2B; mean difference = 22%, *p*-value = 9.12E−37). In females, the CpG site achieving genome-wide significance with the highest degree of hypermethylation was cg22905511 (Fig. 2C; mean difference = − 11%, *p*-value = 2.85E−13), which is located in a CpG island promoter of *C5orf63*. The vast majority (4922; 94.4%) of differentially methylated sites showed a direction of effect consistent with the full dataset in each of the 3 major studies (GSE108567, GSE71678, GSE75248) contributing most of the samples the full term analysis (498 out of 532 samples); an even greater majority of sites (5195; 99.7%) showed a consistent effect in 2 of the 3 major studies (Supplementary Data 1, Supplementary Fig. 6B–C). The majority (3793; 73%) of genome-wide significant sites were hypermethylated in male samples; these male-hypermethylated sites tended to exhibit larger effect sizes than those hypermethylated in females (Fig. 2D). Finally, genome-wide significant CpG sites were enriched in GO pathways involved in keratinization, cell differentiation as well as immune cell migration and chemotaxis (Table 1, Supplementary Data 2). There was very minimal overlap between the identified differentially methylated sites and published 450K-based genome-wide screens of sex-associated DNAm, across a variety of tissue types (Table 2). Though these studies varied in their methodological approach and probe filtering strategies, this result supports the conclusion that the majority of differentially methylated sites identified herein are placenta-specific. Consistent with this notion, our results did overlap well with a previous study^43^ of full term placenta DNAm differences by fetal sex, capturing 146 of the 166 CpG sites identified in this study.

**Table 1:** Significant (FDR *p*-value < 0.05) Gene Ontology pathways enriched in differentially methylated sites from the full term placenta analysis.

GO ID	ONTOLOGY	TERM	FDR *p*-value
*GO:0018149*	BP	Peptide cross-linking	7.37E−14
*GO:0031424*	BP	Keratinization	7.37E−14
*GO:0030216*	BP	Keratinocyte differentiation	4.07E−13
*GO:0001533*	CC	Cornified envelope	1.93E−12
*GO:0070268*	BP	Cornification	2.95E−12
*GO:0009913*	BP	Epidermal cell differentiation	7.78E−12
*GO:0043588*	BP	Skin development	3.64E−08
*GO:0008544*	BP	Epidermis development	1.20E−07
*GO:0045095*	CC	Keratin filament	1.67E−06
*GO:0030855*	BP	Epithelial cell differentiation	2.41E−04
*GO:0060429*	BP	Epithelium development	8.68E−04
*GO:0099512*	CC	Supramolecular fiber	3.50E−03
*GO:0099081*	CC	Supramolecular polymer	4.55E−03
*GO:0099080*	CC	Supramolecular complex	4.55E−03
*GO:0048020*	MF	CCR chemokine receptor binding	1.12E−02
*GO:0048245*	BP	Eosinophil chemotaxis	1.39E−02
*GO:0008009*	MF	Chemokine activity	1.56E−02
*GO:0072677*	BP	Eosinophil migration	1.83E−02
*GO:0048513*	BP	Animal organ development	3.51E−02

**Table 2:** Overlap of significant findings from previous studies of differential DNAm by sex with full term placenta results. Results from previous studies restricted to autosomes.

Study	Year	Tissue	# Significant autosomal CpG sites differential by sex	# Significant sites included in post QC, full term placenta dataset (%)^a^	Overlap with placenta full term results (%)^b^
Price et al	2013	Peripheral blood, adult	45	40 (88.89%)	13 (0.25%)
Hall et al	2014	Pancreatic islets	470	339 (72.13%)	55 (1.06%)
Xu et al	2014	Prefrontal cortex	13,560	13,554 (99.96%)	331 (6.35%)
Inoshita et al	2015	Peripheral leukocytes	292	292 (100%)	103 (1.98%)
Singmann et al	2015	Peripheral blood	11,010	11,008 (99.98%)	530 (10.2%)
Spiers et al	2015	Fetal cortex	521	521 (100%)	103 (1.98%)
Yousefi et al	2015	Cord blood	3031	3031 (100%)	318 (6.10%)
Martin et al	2017	Placenta	15	1 (6.7%)	1 (0.02%)
Suderman et al	2017	Cord blood	11,965	11,961 (99.97%)	274 (5.26%)
		Peripheral blood, 7 years	13,672	13,669 (99.98%)	310 (5.95%)
		Peripheral blood, 15–17 years	12,215	12,214 (99.99%)	291 (5.58%)
Xia et al	2019	Prefrontal cortex, Bulk Brain	15,417	15,048 (97.61%)	439 (8.42%)
Inkster et al	2021	Placenta	166	166 (100%)	146 (2.8%)

^a^Percentage indicates proportion of significant sites from specified study included in placenta full term, post QC dataset.

^b^Percentage indicates proportion of significant sites from specified study found in significant findings from full term analysis (*p* < 1E−8, n = 5212).

Of the 5212 differentially methylated sites, 1285 (24.7%) were identified as distinguishing full term placenta cell types in a recent paper by Yuan et al. examining cell type specific DNAm patterns in term and early term placentas^35^. This subset of cell type distinguishing probes did not appear to differ markedly from those that did not distinguish cell type in the distribution of effect sizes from the fetal sex comparison (Supplementary Fig. 7). However, GO pathway analysis stratified by probe group type revealed that while keratinization and cell differentiation pathways were shared in the two probe groups, the cell type distinguishing probes were uniquely enriched in chemokine and immune cell migration pathways (Supplementary Data 3 and 4).

### Persistence of differentially methylated CpG sites across the gestational period

**Figure 3:**
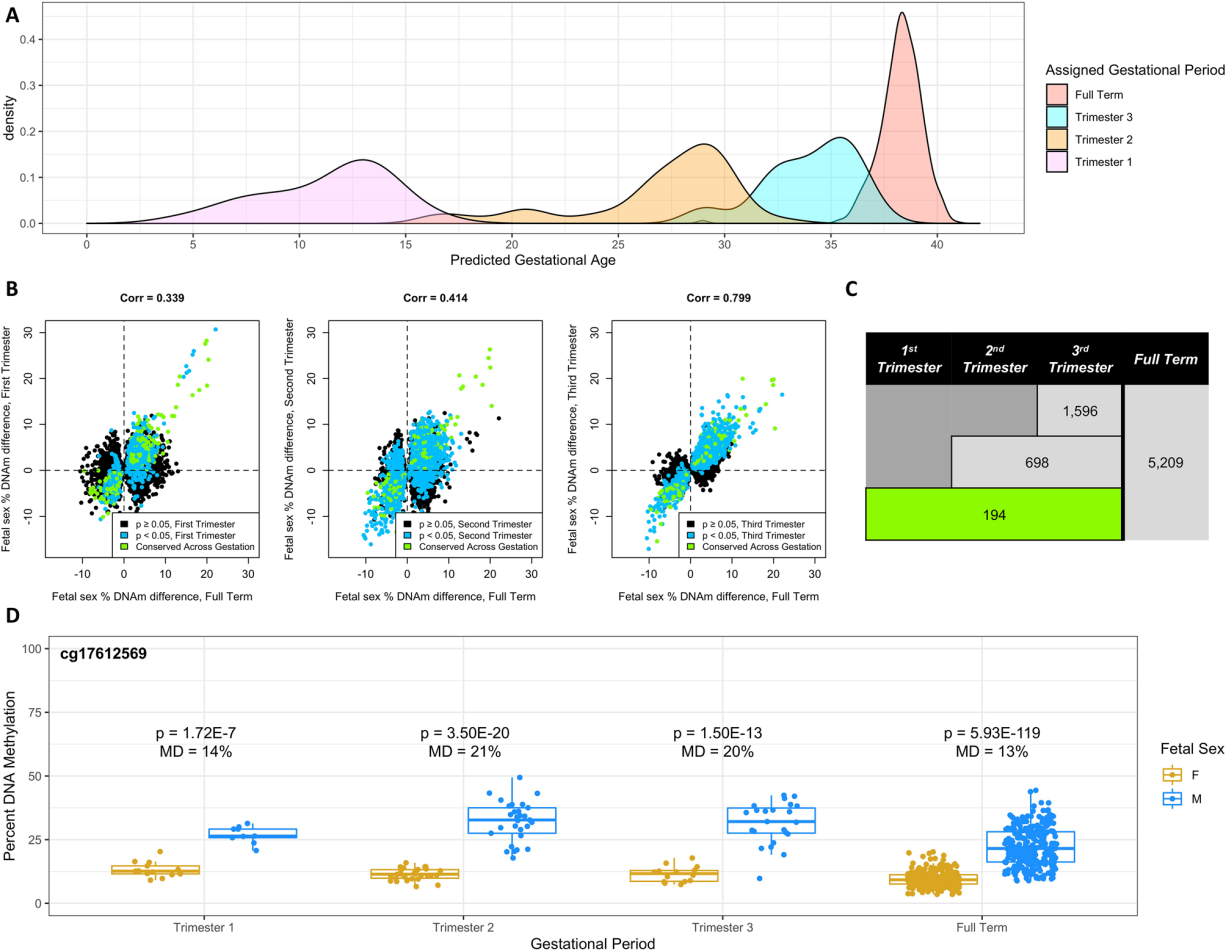
Persistence across the gestational period. (**A**) Distribution of predicted gestational age (via *predictAge()* function from the *planet* R package) in full term and individual trimester datasets (**B**) The persistence correlation analysis shows plots of DNA methylation differences for the three trimesters. The green colored points are the CpG sites that were conserved across gestation. The blue colored points represent the CpG sites that were considered significant for each trimester. The correlation values between the trimester and the full term analysis increase as the full term is approached. (**C**) Persistence of hits discovered from the full term placentas throughout gestation. If the significant site from the full term had a *p* value less than 0.05 and had the same direction of association in the preterm analysis, then it was considered “persistent.” For example, of the 5,209 differentially methylated sites identified in the full term sample that passed QC in all three individual early term samples, 194 CpG sites were persistent per this definition across all three trimesters. (**D**) Comparison of female and male fetuses’ methylation values at different points during gestation. cg17612569 was consistently significant at all time points.

We additionally identified publicly available DNAm samples from preterm placentas, and conducted trimester-specific QC and association analysis using identical methods as used in the full term samples (Supplementary Figs. 2–4; see “Methods” for classification of placentas into these trimesters). The goal of these analyses of early gestation samples was to understand the extent to which differentially methylated sites identified in full term placenta samples persisted across gestation. Gestational age prediction verified that the full term and trimester-specific datasets (N = 22, 59, and 37 for Trimesters 1–3, respectively) captured their intended gestational periods (Fig. 3A). Intuitively, greater correlation was observed in effect sizes as trimesters approached full term; for example the correlation between first trimester and full term effect sizes was 34% while the correlation between third trimester and full term effect sizes was 80% (Fig. 3B). Of the 5209 genome-wide significant CpG sites from the full term analysis (3 CpG sites did not pass QC in ≥ 1 trimester-specific dataset), 194 CpG sites replicated their association with fetal sex in each trimester (Fig. 3C; replication defined as *p*-value ≤ 0.05 and consistent direction of effect as seen in full term samples). An example of one of these cross-gestation persistent CpG sites is cg17612569, which maintains a hypermethylated state in male placenta samples of at least 13% from the first trimester through full term (Table 3, Fig. 3D). Full genome-wide association statistics for the analyses from each trimester have been made available (Supplementary Data 5–7; Supplementary Fig. 8).

**Table 3:** Top 10 CpG sites from full term analysis (ranked by absolute value of effect size in full term analysis) displaying persistence across the gestational period.

CpG site	MD, full term	*P*-value, full term	MD, 3rd trimester	*P*-value, 3rd trimester	MD, 2nd trimester	*P*-value, 2nd trimester	MD, 1st trimester	*P*-value, 1st trimester
cg02375258	20.4	1.46E−31	9.1	0.0387	14	0.00378	24.1	0.000743
cg18237551	20	8.15E−35	19.8	0.000453	22.4	1.02E−05	18.4	0.00035
cg08580836	19.9	9.05E−39	18.6	0.000253	26.3	2.83E−06	28.2	2.03E−05
cg21228005	19.6	3.4E−39	19.6	0.00185	24.5	1.85E−06	27.6	2.1E−06
cg02343823	18.2	3.58E−36	14.4	0.000688	18.6	1.2E−05	17.5	3.02E−05
cg04675542	16.5	6.38E−30	13.3	0.00038	20.4	1.11E−05	16.4	5.69E−05
cg11291313	13.4	1.01E−33	12	0.00164	18.2	5.29E−05	20.4	6.01E−05
cg19014419	13	2.88E−34	13.4	0.000429	17.8	4.48E−05	18.6	0.000183
cg17612569	12.6	5.93E−119	20	1.5E−13	20.7	3.5E−20	13.7	1.72E−07
cg02325951	12.1	5.81E−182	13.4	3.14E−16	11.6	1.5E−12	11.8	1.17E−05

Mean Difference (MD) values calculated as mean in male samples—mean in female samples.

### Region-based differential DNAm analyses and persistence across gestation

**Figure 4:**
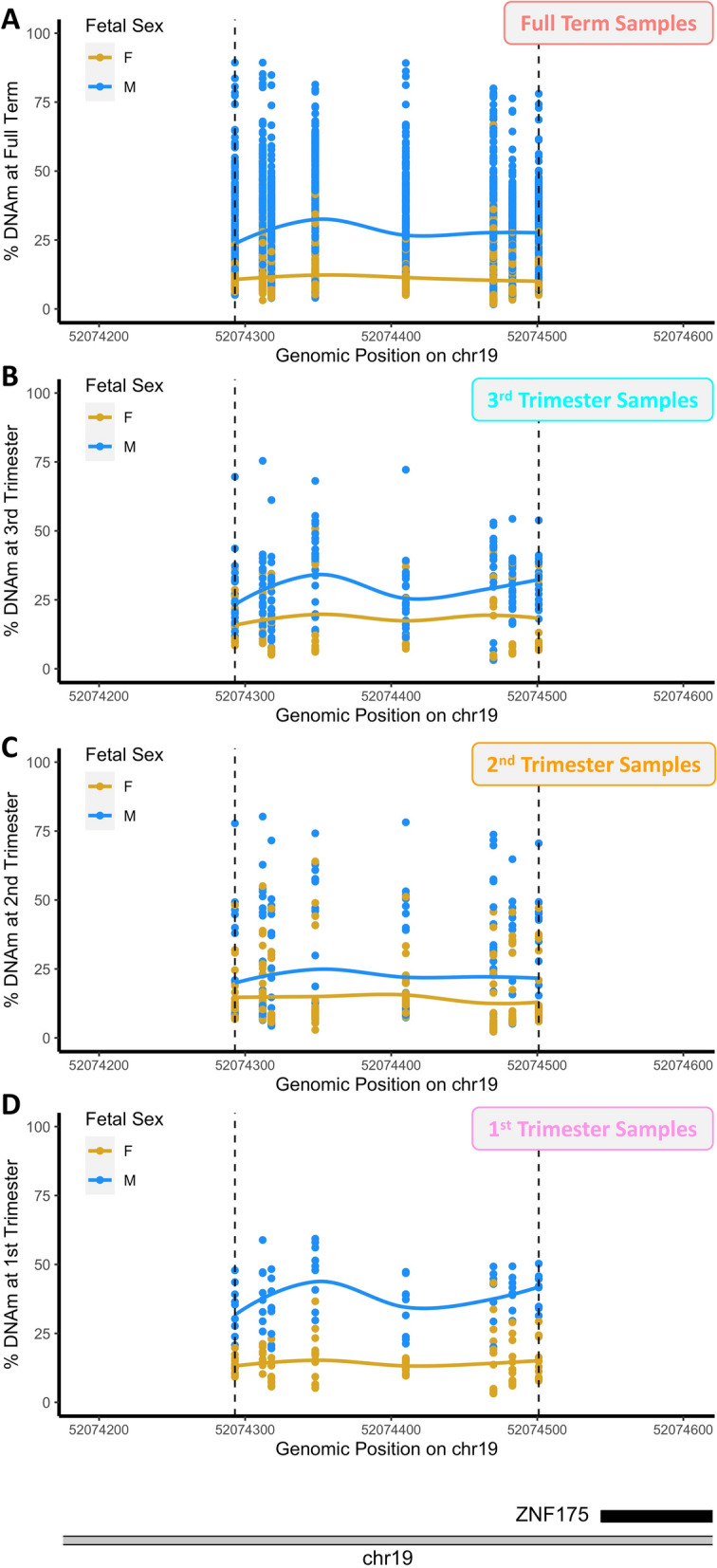
DMR at the *ZNF175* promoter across the gestational period. Top-ranked DMR from full term analysis at *ZNF175* promoter. DMRs are plotted as percent methylation as a function of genomic position. Dots indicate samples and solid lines indicate smooth lines through male sample values (blue) and female sample values (gold). Actual identified DMR region indicated via dashed lines. (**A**) Full Term Samples. (**B**) 3rd Trimester Samples (**C**) 2nd Trimester Samples (**D**) 1st Trimester Samples.

We also sought to identify DMRs in full term samples and examine the extent to which they persisted across gestation. Nine DMRs were identified as significant beyond a discovery-based threshold (fwerArea value) of 0.1 (Supplementary Fig. 9, Supplementary Data 8). The top-ranked DMR was located in a CpG island promoter of *ZNF175* (fwerArea = 0); males exhibited 17% higher average DNAm relative to females in this region (Fig. 4A). Trimester-specific region-based analyses (Supplementary Data 9–11) were not statistically powered for genome-wide detection. However, these analyses did reveal that the identified *ZNF175* promoter region exhibited a similar degree of hypermethylation in males across the gestational period (Fig. 4B–D). In fact, all of the nine full term DMRs exhibited evidence for persistence across gestation via consistent direction of effect across all trimesters (Table 4, Supplementary Figs. 10–17). However, in most cases there was a significantly decreased magnitude of effect size in the individual trimesters. The most robust evidence for persistence, beyond the *ZNF175* DMR, was observed for male hypermethylation at *ZNF300* promoter (Supplementary Fig. 10) and for female hypermethylation at the *C5orf63* promoter (Supplementary Fig. 11). In the latter case, the DMR did not emerge until the 2nd trimester, but persisted thereafter at a similar magnitude.

**Table 4:** Persistence of genome-wide significant differentially methylated regions from full term analysis across the gestational period.

chr	start	end	*p*. value	fwer	*p*. value area	fwer area	MD, full term	Nearest gene (location)	MD, trimester1	MD, trimester 2	MD, trimester 3	Contains cell type discriminating probes?
chr19	52,074,293	52,074,501	0	0	0	0	0.17	*ZNF175* (promoter)	0.24	0.0831	0.11	No
chr5	150,284,416	150,284,796	0	0	0	0	0.172	*ZNF300* (promoter)	0.21	0.212	0.159	Yes
chr5	126,408,756	126,409,553	0	0	0	0	− 0.0737	*C5orf63* (promoter)	− 0.00439	− 0.101	− 0.101	Yes
chr11	2,906,285	2,907,127	0	0	1.83E−06	0.007	0.0243	*CDKN1C* (gene body)	0.0325	0.0159	0.0295	No
chr7	86,974,674	86,975,244	2.61E−07	0.001	3.66E−06	0.014	− 0.0522	*CROT* (promoter)	− 0.0514	− 0.0743	− 0.0761	Yes
chr7	27,169,674	27,171,051	5.23E−07	0.002	1.05E−05	0.039	− 0.0372	*HOXA4* (promoter)	− 0.0106	− 0.0035	0.00492	Yes
chr4	90,758,120	90,759,203	1.05E−06	0.004	1.73E−05	0.065	− 0.0393	*SNCA* (promoter)	− 0.0216	− 0.0138	− 0.00186	No
chr6	28,641,622	28,642,394	3.4E−06	0.013	1.83E−05	0.069	0.0329	*ZBED9* *(58 Kb downstream*	0.0419	0.0692	0.0452	Yes
chr9	98,637,332	98,638,444	2.61E−07	0.001	2.25E−05	0.085	− 0.0561	*ERCC6L2* (promoter)	− 0.0427	− 0.0143	− 0.0321	No

Statistics generated from *bumphunter()*^64^ function in *minfi* package^57^. Mean Difference (MD) values calculated as mean in male samples—mean in female samples. Final column indicates if DMR contained cell type distinguishing probe as identified by Yuan et al. in full term samples.

**Figure 5:**
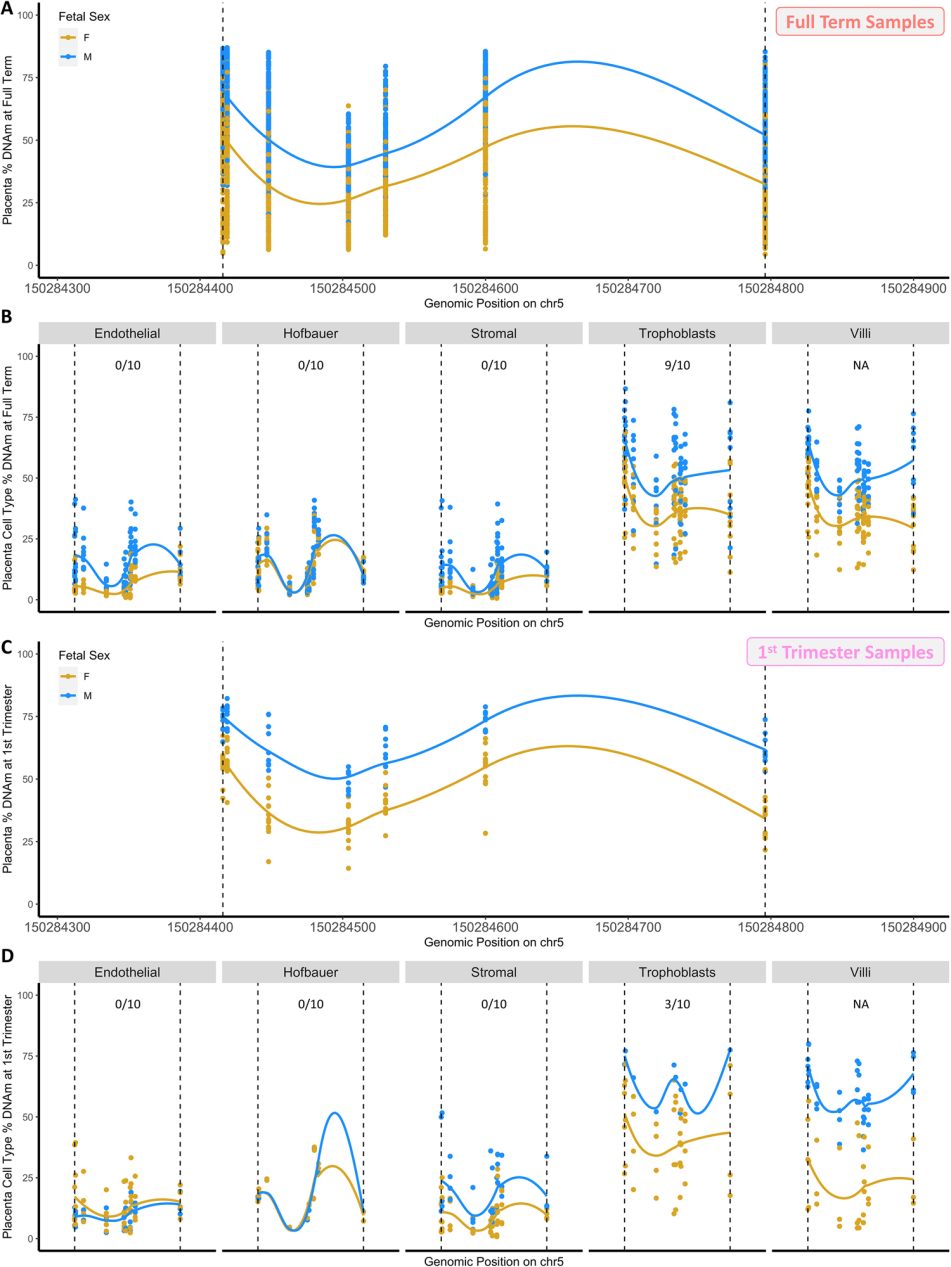
Cell type heterogeneity at the *ZNF300* promoter. DMRs are plotted as percent methylation as a function of genomic position. Dots indicate samples and solid lines indicate smooth lines through male sample values (blue) and female sample values (gold). Actual identified DMR region indicated via dashed lines. (**A**) DMR identified at *ZNF300* promoter on chromosome 5 in full term analysis, plotted using full term samples from present study. (**B**–**D**) The same region, but plotted using samples from different datasets. (**B**) Term samples and individual placenta cell types from Yuan et al. (**C**) 1st Trimester samples from present study. (**D**) 1st trimester samples and individual placenta cell types from Yuan et al. In b + d, fractions indicate the proportion of probes in the region that were annotated by Yuan et al. as distinguishing that cell type in full term and 1st trimester samples, respectively. Villi samples were not evaluated in this manner by Yuan et al. as this is the unsorted bulk tissue.

Of the nine DMRs, five contained probes that were identified as distinguishing cell type by Yuan et al. (Table 4). Plotting the placenta cell type specific DNAm patterns from this study in the regions defining these DMRs revealed in several instances that particular cell types drive the fetal sex DNAm patterns we observed. For example, male hypermethylation in the *ZNF300* promoter is primarily driven by sex differences in trophoblast cell types in this region (Fig. 5A–B). Just as we observed this fetal sex differential DNAm to persist throughout gestation (Fig. 5C, Table 4, Supplementary Fig. 10), the trophoblast signatures remain when examining the 1st trimester samples from Yuan et al. (Fig. 5D). For the remaining four DMRs containing cell type distinguishing probes, cell type-related inferences are less clear (Supplementary Figs. 18–21), likely owing to the smaller magnitude of DNAm difference in the DMRs. Nonetheless, it can be observed that sex differences in trophoblast DNAm contribute to the DMRs observed at the *C5orf63* (Supplementary Fig. 18) and *CROT* (Supplementary Fig. 19) promoters, with endothelial cells playing a role in the latter case as well, but only in the early gestation time point.

### Differential expression analysis of genes implicated by DMRs

**Figure 6:**
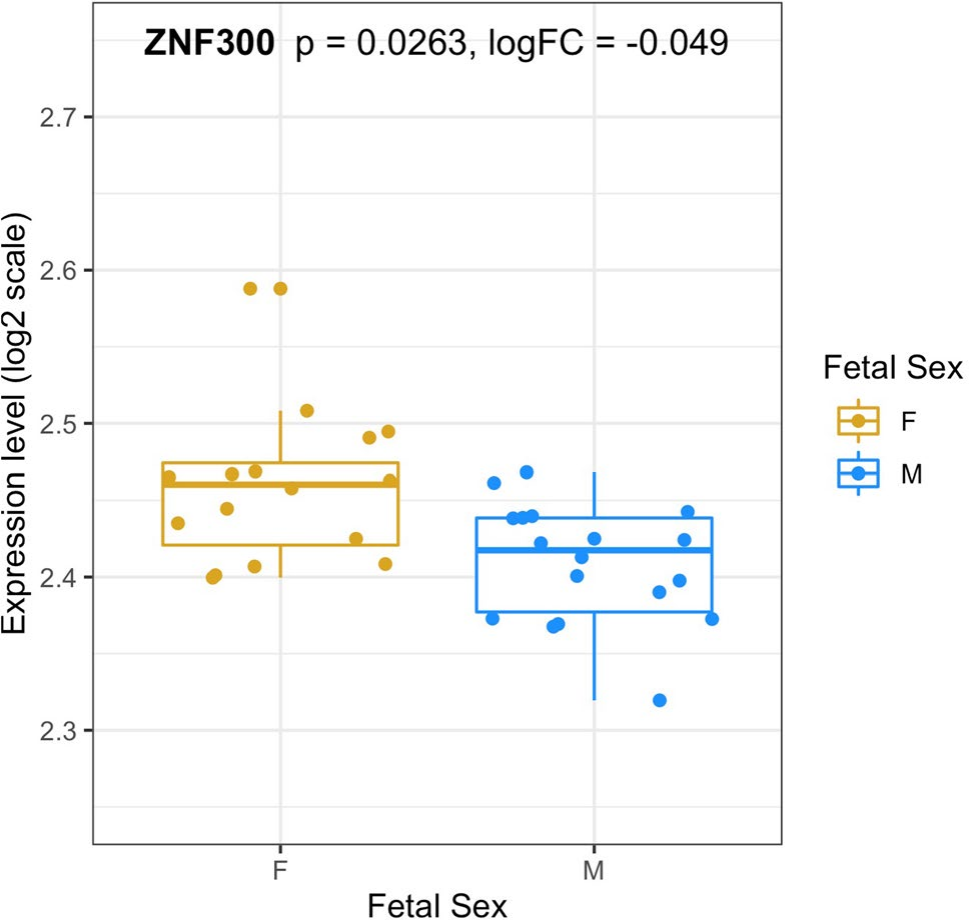
Placenta gene expression at ZNF300 Box plots of the distribution of *ZNF300* gene expression (log2 scale) in males (blue) and females (gold) represented in the GSE75010 dataset.

Finally, we examined if the genes implicated by the nine significant DMRs from the full term analysis (Table 4) showed evidence for differential placenta gene expression using microarray data from a single publicly available dataset (GSE75010; paired DNAm samples from GSE98224 were analyzed in this study, see Supplementary Table 1). We observed evidence for differential *ZNF300* expression (log2 fold change = − 0.049; *p* = 0.0263) with males showing reduced expression, the direction of effect expected due to the hypermethylation in males at the *ZNF300* promoter observed in this study (Fig. 6). We did not observe evidence for differential expression at any of the other 8 DMRs from the full term analysis (Supplementary Table 2).

## Discussion

Here we present the largest study to date examining placental DNAm differences by fetal sex. Both single site and region-based analyses revealed large differences according to fetal sex in the placenta methylome. Single site analysis of full term placenta samples demonstrated that a total of 5212 CpG sites were significantly associated with fetal sex at a genome-wide significance threshold of 1E-8. Pathway analysis results showed that some of the most significantly enriched biological processes were keratinization, cell differentiation, and eosinophil chemotaxis. Replication studies using samples at earlier points in gestation demonstrated evidence that 194 of these CpG sites are differentially methylated by sex throughout pregnancy. Nine DMRs met a discovery-based threshold of significance, and the top-ranked DMRs at the promoters of *ZNF175* and *ZNF300*, respectively, displayed robust evidence for persistence across the gestational period. In the latter case, cell type specific placenta DNAm data revealed that trophoblast cell types drive the fetal sex association observed in the region.

Our findings of many differentially methylated sites and regions according to fetal sex will contribute to the understanding of the molecular mechanisms driving sex-specific placenta structure and functions. For example, several immune-related pathways were enriched in differentially methylated CpG sites from the full term analysis, which is consistent with several previous investigations of sex-related gene expression differences in placenta^37,39,40^. A recent dataset described by Yuan et al.^35^, which profiled DNAm in sorted placenta cell types, allowed for further characterization of these immune pathway findings. While the Yuan et al. study found no significant differences in estimated cell type proportions between male and female placentas, it is still possible that individual probes exhibit both sex-specific and cell type specific DNAm patterns. Indeed, we found that a significant proportion (1285, 24.7%) of our 5212 fetal sex differentially methylated sites in full term placentas were defined by Yuan et al. to distinguish placenta cell types at full term as well. Stratified pathway analyses considering probes in this group of 5212 that did and did not distinguish placenta cell types separately revealed that the enrichment of chemokine and immune cell migration pathways seen in the overall group of probes was observed uniquely in the cell-type-distinguishing subset. This result argues that sex-specific immune responses in the placenta are mediated through cell type proportion changes.

Keratinization and cell differentiation pathways were also strongly enriched in the differentially methylated sites at full term, and were enriched in both the cell type distinguishing and non-cell-type-distinguishing probe subsets separately. Similar pathways were found in enrichment studies from a previous investigation of gene expression differences by fetal sex^38^. In addition, the top 4-ranked pathways in our study, including “peptide cross-linking” and keratinocyte-related pathways, were also previously found to be enriched in a gene set consisting of “core” placenta genes that are conserved across evolution and described as “central for making a placental mammal”^45^, suggesting that sex-specific DNAm in the placenta affects genes that are fundamental to the placenta’s function.

A unique feature of our analysis was the exploration of placenta samples from earlier time points in pregnancy. Though these datasets were not conducive to genome-wide discovery owing to their small sample sizes, they did provide an ability to infer which CpG sites and regions identified as differentially methylated in full term samples displayed similar patterns of DNAm throughout the gestational period. We identified 194 CpG sites that met p-value thresholds of differential DNAm, with consistent direction of effect, in all four analyzed datasets (1st Trimester through full term). As placenta datasets continue to be generated, it will be increasingly possible to better characterize persistently differentially methylated sites by fetal sex, to a greater degree possible than this study. Identification of these specific sites (and the genes and pathways they implicate) will be necessary to gain a greater understanding of the placenta’s role in “programming” sex-discordant phenotypes throughout the life course^3–5^.

At the region level, the two top-ranked DMRs from the full term analysis showed robust evidence for persistence across the gestational period. The top-ranked DMR, located in the CpG-island promoter of *ZNF175*, is characterized by a 17% hypermethylation in male samples relative to female, implying greater *ZNF175* gene expression in female samples. *ZNF175* regulates the expression of several chemokine receptors, and has been previously demonstrated to be up-regulated in pre-eclamptic vs. healthy placentas^23,24^. However, these studies not only examined the maternal portion of the tissue but also were limited by very small sample sizes, leaving much to be understood about the role of *ZNF175* in the fetal placenta. Future studies should aim to validate the impact of *ZNF175* promoter DNAm on *ZNF175* expression, characterize the impact of *ZNF175* modulation in placenta (trophoblast) cells directly, and dissect the potential role of *ZNF175* promoter DNAm in fetal placenta in the context of adverse pregnancy outcomes and their interaction with fetal sex.

The second-ranked DMR at the *ZNF300* promoter also displayed male hypermethylation to a similar degree (17%), along with strong evidence for the persistence of this DNAm difference across the gestational period. *ZNF300* is a transcriptional repressor with multiple reported targets^46^. It has been linked to immune cell differentiation^47,48^ and tumorigenesis^49,50^, furthering the longstanding narrative noting the similarity in placenta cells and cancer cells with respect to their invasive properties^51^. Along these lines, we found through incorporation of the Yuan et al. placenta cell type data^35^ that fetal sex differences in *ZNF300* promoter DNAm are driven by differences seen in the trophoblast cell type, which is primarily responsible for placental invasion^52^.

In placenta specifically, this region has previously been associated with fetal sex in placenta (albeit in a study using many of the same public datasets used herein)^43^. Also, it has been previously demonstrated that samples collected from twins discordant for intrauterine growth restriction (IUGR) showed significant hypermethylation at the *ZNF300* promoter in IUGR samples^30^. Our companion paper expands this story further through integration of imputed genotyping and placenta morphology measurements with comprehensive DNAm profiling (whole genome bisulfite sequencing, WGBS)^53^. Specifically, this study also demonstrates the existence of hypermethylation in male placentas at the *ZNF300* promoter, but further shows that these DNAm signatures mediate *cis* and *trans* (X chromosome) effects of genetic variants on placenta area. Collectively, these findings demonstrate robust evidence for a fetal sex DMR in the *ZNF300* promoter that is present across pregnancy and driven by trophoblast cell types, and provide a molecular mechanism for the governance of placenta size by fetal genetic variation. Future (in vitro) work should aim to further investigate the directionality of these phenomena in trophoblast cells specifically, to confirm if CpG island promoter hypermethylation leads to reduced *ZNF300* expression (as is expected canonically and as we observed in a single study lookup) and the relationship between *ZNF300* expression and cell proliferation.

There are several limitations of our approach that need to be recognized. First, we strictly leveraged publicly available data, which have limited annotation. In some cases, we were able to perform quality control or sanity checks to address these shortcomings. For example we used SNP probes on the Illumina 450K Array to identify and remove duplicate samples that may have been submitted publicly more than once, and we used a gestational age prediction algorithm to confirm that our sample classification was consistent with that guided by available metadata. But in some cases these checks were not possible; for example when restricting to samples from normal/typical pregnancies, it was only possible to rely on the provided metadata or details in the manuscript associated with the data, which provided sufficient detail in most cases (Supplementary Table 1) but could often be incomplete. Therefore, we cannot completely exclude the possibility that non phenotypically normal samples entered into our study, in particular for the 1st and 2nd trimester samples. In addition, though we aimed to gather all publicly available 450K placenta data, few early term samples exist. Therefore, these samples were only suitable for lookup for the sites and regions identified in the full term cohort, rather than being powered for genome-wide discovery. Another limitation was the use of datasets that used the 450K array, as newer technologies to query DNAm now exist. Our companion paper^53^ describes an interrogation of sex-related DNAm patterns via WGBS, a comprehensive query of the methylome, but it is currently not feasible to perform these measurements on a large sample size. A better balance of methylome coverage and sample throughput is struck with the next iteration of the 450K Array, the Methylation EPIC array. Finally, our study utilized bulk placenta samples, which are a mixture of placenta cell types. We did perform several post hoc analyses to disentangle the role of different cell types in our findings using placenta DNAm data from sorted cell populations. However, our findings could be strengthened by evaluating fetal sex differences via histologically-determined placenta composition^54^. Additional future work should aim to interrogate fetal sex DNAm differences in individual placenta cell populations directly (instead of as follow-up to bulk placenta findings) to better understand the extent to which different cell types drive sex-related DNAm differences in the placenta.

Overall, future work should seek to better understand the mechanism and consequence of fetal sex DNAm differences by establishing connections with additional molecular, phenotypic, and morphological endpoints. For example, the presence of metadata fields for some datasets and not others in this study limited the potential for sensitivity analyses examining the effect of additional variables of interest, such as birth weight or maternal lifestyle factors, on fetal sex-based DNAm differences. Future studies can look to explicitly measure these factors and examine their impact on fetal sex differences. In addition, paired placenta DNAm and gene expression or simply better integration of these two data types can be used to translate DNAm differences into gene expression changes, particularly in genomic regions outside of CpG island promoters and gene bodies where expectations of these relationships are less well known. While we did observe significant differential placenta gene expression differences at *ZNF300*, there was no significant difference in expression between placentas of male and female newborns for the other 8 genome-wide significant differentially methylated genes/regions. As only one gene expression dataset was used in this analysis, we may be limited in power to find significant differences in expression of these other regions identified by the DNA methylation analysis and interrogating gene expression differences by fetal sex in larger datasets remains an important future area of research. Integration with and generation of additional placenta-specific molecular information, in particular chromatin-level data, will also be helpful in elucdiating the mechanism of the observed placenta DNAm differences by fetal sex. Understanding how these types of data themselves also differ by sex is also a key area of future work. Also, placenta DNAm studies of pregnancy outcomes should be fetal sex-stratified or evaluate an interaction with fetal sex, given the significant differences by fetal sex identified in this study. In a similar vein, studies that collect placenta samples should aim to follow-up subjects throughout childhood and into adulthood whenever possible, to better quantify the potential for fetal sex DNAm differences to “program” sex-discordant health outcomes. Finally, as in our companion paper, future placenta studies should aim to collect placental morphology phenotypes in order to relate observed DNAm changes to placenta size, shape, and structure.

In conclusion, this study presents profound differences in DNAm from full term placentas according to fetal sex at the site and region level. Many of these differences, including strong hypermethylated signals in male samples relative to females at the *ZNF175* and *ZNF300* promoters, show strong evidence for their existence throughout pregnancy. Further characterization of these DNAm signatures will enable improved understanding of sex-specificity in placenta structure and function, gestational environments, and health outcomes across the life course.

## Methods

### Study selection

We searched the Gene Expression Omnibus (GEO) database on July 19th, 2018 for Illumina 450K microarray data sets containing human placental tissue samples by applying the following search terms: “placental methylation” or “placenta methylation” and limiting to platform types of “methylation profiling by array”, “methylation profiling by genome tiling array”, and “methylation profiling by SNP array”. We manually reviewed the resulting 78 studies and removed any that did not use the 450K array or did not make the raw .idat files available. We also restricted our study samples to only those labeled as phenotypically normal or controls whenever possible via the available metadata in GEO or details in the manuscript associated with the data. To assign samples within each study to gestational age (GA) categories of full term, first trimester, second trimester, and third trimester, we used the gestational age variable included in the sample metadata in GEO. Specifically, the first trimester group was defined as GA < 14 weeks, the second trimester as 14 δ GA < 28 weeks, the third trimester as 28 δ GA < 37 weeks, and full term as GA > 37 weeks. If a GA variable was not available, we consulted the manuscript associated with the GEO dataset. A total of 14 GEO datasets comprising 783 samples were included in the analysis, including 637 full term, 33 1st trimester, 72 2nd trimester, and 41 3rd trimester samples (Supplementary Table 1). We predicted the gestational age of these samples using the *predictAge()* function from the planet R package^55^ to verify that our sample groups captured their intended points during the gestation period^56^.

### Array pre-processing, quality control, and batch effect correction

All analyses were performed using R (v4.0.3) and the *minfi* R package (v1.36)^57^ unless otherwise stated. We theorized that some of the placenta samples in the public domain could be duplicates. Hence, from the samples that passed the inclusion criteria, duplicate pairs of samples were identified using the 65 single nucleotide polymorphisms (SNP) probes measured on the 450K array. The sample from the identified pair with the greater number of probes defined as detection p-value failures (*p* > 0.01) was removed. The *preprocessNoob()* function^58,59^ was used for background correction and dye-bias equalization. Samples that had low overall intensity (median unmethylated or methylated signal < 11) or had a detection *p*-value > 0.01 in more than 1% of probes or probes that had a detection *p*-value > 0.01 in more than 10% of samples were removed. Samples were removed if the reported sex did not match predicted sex generated by the *getSex()* function in *minfi*. We removed probes mapping to sex chromosomes, and also removed autosomal-targeting probes that ambiguously map to sex chromosome locations according to Chen et al.^60^. After these steps the dataset for the full term samples consisted of 445,278 probes and 532 samples. Finally, quantile normalization was performed, and surrogate variables were estimated using the sva R package^61^ on the resulting M-values. SVs have been shown to capture differences related to batch effects and cell type proportions across samples in a wide variety of simulated settings^62^, and to remove the effects of unwanted sources of technical and biological variation^61^.

We repeated these QC and batch effect correction steps in each trimester dataset individually. For the 1st trimester dataset, these steps resulted in 444,948 probes and 22 samples available for downstream analysis. For the 2nd trimester, it was 445,331 probes and 59 samples and for the 3rd trimester it was 445,220 probes and 37 samples.

### Identification of differentially methylated CpG sites and regions

To identify differentially methylated individual CpG sites using the *limma* R package^63^, we modeled M-values as function of fetal sex and the estimated SVs and defined genome-wide significant probes as those with *p*-values < 1E-8. The *bumphunter()* function^64^ was used to identify differentially methylated regions (DMRs) using the same model; p-values were assessed via a bootstrapping approach that performed 1000 resamples of the data. Please see the *bumphunter()* documentation for additional details. We repeated these same association testing procedures in each trimester individually. Because these datasets were not powered for genome-wide discovery, we did not impose strict statistical significance cutoffs for these analyses. Instead, we looked for evidence of replication of differentially DNAm identified in the full term samples, to quantify the extent to which these differences persisted across the gestational period. We considered a early term probe to replicate the full term result (i.e. was “persistent”) if the fetal sex analysis showed a *p*-value < 0.05 and a consistent direction of effect with that observed in the full term samples.

### Gene enrichment and pathway analysis

After identifying differentially methylated CpG sites and DMRs, we sought to characterize biological pathways enriched in our top findings. All probes reaching a significance threshold of 1E-8 were evaluated for enrichment in Gene Ontology (GO) pathways using the *gometh()* and *topGSA()* functions in the R package *missMethyl*^65^.

### Comparison of results to previous DNAm studies of sex

Significant CpG sites discovered via our analysis were compared to significant CpG sites found in previous human sex-related DNAm studies to determine how many sites had already been identified in the literature. These previous studies analyzed placenta^41,43^ as well as cord blood^66,67^, peripheral blood^67–70^, prefrontal cortex^71,72^, pancreas^73^, and fetal cortex^74^. Of note, several public datasets utilized by Inkster et al.^43^ were also used in this current study.

### Cell type heterogeneity analyses

To understand the extent to which our fetal sex differentially methylated sites and regions were driven by cell type heterogeneity, we compared our results to those from a recent study by Yuan et al. examining placenta cell type-specific DNAm patterns^35^. Specifically, we quantified the degree of overlap in differentially methylated sites from our full term analysis with probes identified in the Yuan et al. study as distinguishing cell type at full term, and conducted GO pathway analyses in groups of probes that did and did not overlap with this list separately. We also determined which of the identified DMRs from the full term analysis contained probes from this list, downloaded the data from the Yuan et al. study (GEO ID: GSE159526) and plotted the cell type specific DNAm profiles in these same regions to determine which cell type(s) were driving the DMR result.

### Differential expression analysis

We used publicly available human placental microarray gene expression dataset (GEO ID: GSE75010) which consists of 157 highly annotated placental samples. Of these 157 samples, we excluded those from deliveries that were preterm or associated with pre-eclampsia for our transcriptomics analysis. To assess the normalization of the 34 remaining term placental samples, we used the *exprs* function of the Biobase R package to retrieve the expression values, then performed a log_2_ transformation and created boxplots to visualize if the data had been normalized. We allocated the samples in our dataset to our groups of interest (18 male, 16 female), then used the *limma* R package to determine the differential expression of genes between placentas of male and female newborns. A significance threshold of 0.05 was applied to Benjamini-Hochberg–corrected p–values. We also generated boxplots of the distribution of gene expression for the top genome-wide significant differentially methylated genes/regions.

## Supplementary Information

Supplementary Information 1.

Supplementary Information 2.

Supplementary Information 3.

Supplementary Information 4.

Supplementary Information 5.

Supplementary Information 6.

Supplementary Information 7.

Supplementary Information 8.

Supplementary Information 9.

Supplementary Information 10.

Supplementary Information 11.

Supplementary Information 12.

Supplementary Table and Figures.

## Data Availability

All placenta datasets used for association testing in this study are available in GEO and listed in Supplementary Table 1. Cell type specific placenta DNAm data described in Yuan et al.^35^ were also downloaded from GEO (GSE159526). Placenta gene expression data was also downloaded from GEO (GSE75010).

## Code availability

Scripts for analyses conducted in this study are available at https://github.com/sandrews5/PlacentaDNAm_FetalSex.

## References

[1] Burton GJ, Fowden AL (2015). The placenta: A multifaceted, transient organ. Philos. Trans. R. Soc. B Biol. Sci..

[2] Bianco-Miotto T (2016). Recent progress towards understanding the role of DNA methylation in human placental development. Reprod. Camb. Engl..

[3] Barker DJP (2004). The developmental origins of well-being. Philos. Trans. R. Soc. Lond. B Biol. Sci..

[4] Gillman MW (2005). Developmental origins of health and disease. N. Engl. J. Med..

[5] Novakovic B, Saffery R (2012). The ever growing complexity of placental epigenetics—role in adverse pregnancy outcomes and fetal programming. Placenta.

[6] Werling DM, Geschwind DH (2013). Sex differences in autism spectrum disorders. Curr. Opin. Neurol..

[7] Aleman A, Kahn RS, Selten J-P (2003). Sex differences in the risk of schizophrenia: Evidence from meta-analysis. Arch. Gen. Psychiatry.

[8] Ngo ST, Steyn FJ, McCombe PA (2014). Gender differences in autoimmune disease. Front. Neuroendocrinol..

[9] Cogswell ME, Yip R (1995). The influence of fetal and maternal factors on the distribution of birthweight. Semin. Perinatol..

[10] Wallace JM, Bhattacharya S, Horgan GW (2013). Gestational age, gender and parity specific centile charts for placental weight for singleton deliveries in Aberdeen, UK. Placenta.

[11] Bracero LA, Cassidy S, Byrne DW (1996). Effect of gender on perinatal outcome in pregnancies complicated by diabetes. Gynecol. Obstet. Invest..

[12] Bekedam DJ, Engelsbel S, Mol BWJ, Buitendijk SE, van der Pal-de Bruin KM (2002). Male predominance in fetal distress during labor. Am. J. Obstet. Gynecol..

[13] Zeitlin, J., Ancel, P.-Y., Larroque, B., Kaminski, M., & EPIPAGE Study. Fetal sex and indicated very preterm birth: Results of the EPIPAGE study. *Am. J. Obstet. Gynecol.* **190**, 1322–1325 (2004). 10.1016/j.ajog.2003.10.703 15167836

[14] Cui W (2005). Sex differences in birth defects: a study of opposite-sex twins. Birth Defects Res. A. Clin. Mol. Teratol..

[15] Peelen MJCS (2016). Impact of fetal gender on the risk of preterm birth, a national cohort study. Acta Obstet. Gynecol. Scand..

[16] Challis J, Newnham J, Petraglia F, Yeganegi M, Bocking A (2013). Fetal sex and preterm birth. Placenta.

[17] Vatten LJ, Skjaerven R (2004). Offspring sex and pregnancy outcome by length of gestation. Early Hum. Dev..

[18] Murphy VE (2003). Maternal asthma is associated with reduced female fetal growth. Am. J. Respir. Crit. Care Med..

[19] Stark MJ, Clifton VL, Wright IMR (2009). Neonates born to mothers with preeclampsia exhibit sex-specific alterations in microvascular function. Pediatr. Res..

[20] Murphy VE, Gibson P, Talbot PI, Clifton VL (2005). Severe asthma exacerbations during pregnancy. Obstet. Gynecol..

[21] Clifton VL (2010). Review: Sex and the human placenta: Mediating differential strategies of fetal growth and survival. Placenta.

[22] Clark JM (2007). Effect of maternal asthma on birthweight and neonatal outcome in a British inner-city population. Paediatr. Perinat. Epidemiol..

[23] Trifonova EA (2014). Analysis of the placental tissue transcriptome of normal and preeclampsia complicated pregnancies. Acta Nat..

[24] Kaartokallio T, Cervera A, Kyllönen A, Laivuori K (2015). Gene expression profiling of pre-eclamptic placentae by RNA sequencing. Sci. Rep..

[25] Li J (2015). A MicroRNA signature in gestational diabetes mellitus associated with risk of macrosomia. Cell. Physiol. Biochem..

[26] Majewska M (2019). Placenta transcriptome profiling in intrauterine growth restriction (IUGR). Int. J. Mol. Sci..

[27] Tilley SK (2018). Placental CpG methylation of infants born extremely preterm predicts cognitive impairment later in life. PLoS ONE.

[28] Schroeder DI (2016). Placental methylome analysis from a prospective autism study. Mol. Autism.

[29] Leavey K, Wilson SL, Bainbridge SA, Robinson WP, Cox BJ (2018). Epigenetic regulation of placental gene expression in transcriptional subtypes of preeclampsia. Clin. Epigenetics.

[30] Roifman M (2016). Genome-wide placental DNA methylation analysis of severely growth-discordant monochorionic twins reveals novel epigenetic targets for intrauterine growth restriction. Clin. Epigenetics.

[31] Scott NM (2009). Placental cytokine expression covaries with maternal asthma severity and fetal sex. J. Immunol. Baltim. Md.

[32] Scott NM (2011). The presence of maternal asthma during pregnancy suppresses the placental pro-inflammatory response to an immune challenge in vitro. Placenta.

[33] Osei-Kumah A, Smith R, Jurisica I, Caniggia I, Clifton VL (2011). Sex-specific differences in placental global gene expression in pregnancies complicated by asthma. Placenta.

[34] Jaffe AE, Irizarry RA (2014). Accounting for cellular heterogeneity is critical in epigenome-wide association studies. Genome Biol..

[35] Yuan V (2021). Cell-specific characterization of the placental methylome. BMC Genom..

[36] Wang Y, Zhao S (2010). Vascular Biology of the Placenta.

[37] Cvitic S (2013). The human placental sexome differs between trophoblast epithelium and villous vessel endothelium. PLoS ONE.

[38] Buckberry S, Bianco-Miotto T, Bent SJ, Dekker GA, Roberts CT (2014). Integrative transcriptome meta-analysis reveals widespread sex-biased gene expression at the human fetal-maternal interface. Mol. Hum. Reprod..

[39] Gonzalez TL (2018). Sex differences in the late first trimester human placenta transcriptome. Biol. Sex Differ..

[40] Sood R, Zehnder JL, Druzin ML, Brown PO (2006). Gene expression patterns in human placenta. Proc. Natl. Acad. Sci. U.S.A..

[41] Martin E (2017). Sexual epigenetic dimorphism in the human placenta: implications for susceptibility during the prenatal period. Epigenomics.

[42] Gong S (2018). Genome-wide oxidative bisulfite sequencing identifies sex-specific methylation differences in the human placenta. Epigenetics.

[43] Inkster AM (2021). A cross-cohort analysis of autosomal DNA methylation sex differences in the term placenta. Biol. Sex Differ..

[44] Eisenhauer JG (2021). Meta-analysis and mega-analysis: A simple introduction. Teach. Stat..

[45] Dunwell TL, Paps J, Holland PWH (2017). Novel and divergent genes in the evolution of placental mammals. Proc. R. Soc. B Biol. Sci..

[46] Qiu H (2008). Identification of the DNA binding element of the human ZNF300 protein. Cell. Mol. Biol. Lett..

[47] Xu J-H (2010). PU.1 can regulate the ZNF300 promoter in APL-derived promyelocytes HL-60. Leuk. Res..

[48] Cai J (2014). ZNF300 knockdown inhibits forced megakaryocytic differentiation by phorbol and erythrocytic differentiation by arabinofuranosyl cytidine in K562 cells. PLoS ONE.

[49] Wang T (2012). Overexpression of the human ZNF300 gene enhances growth and metastasis of cancer cells through activating NF-kB pathway. J. Cell. Mol. Med..

[50] Yu S (2020). ZNF300 promotes chemoresistance and aggressive behaviour in non-small-cell lung cancer. Cell Prolif..

[51] Murray MJ, Lessey BA (1999). Embryo implantation and tumor metastasis: Common pathways of invasion and angiogenesis. Semin. Reprod. Endocrinol..

[52] Knöfler M (2019). Human placenta and trophoblast development: Key molecular mechanisms and model systems. Cell. Mol. Life Sci. CMLS.

[53] Ladd-Acosta, C. *et al.* Placenta DNA methylation at ZNF300 is associated with fetal sex and placental morphology. *bioRxiv* 2021.03.05.433992. 10.1101/2021.03.05.433992 (2021).

[54] Mayhew TM, Wadrop E, Simpson RA (1994). Proliferative versus hypertrophic growth in tissue subcompartments of human placental villi during gestation. J. Anat..

[55] Yuan V (2019). Accurate ethnicity prediction from placental DNA methylation data. Epigenetics Chromatin.

[56] Lee Y (2019). Placental epigenetic clocks: Estimating gestational age using placental DNA methylation levels. Aging.

[57] Aryee MJ (2014). Minfi: A flexible and comprehensive Bioconductor package for the analysis of Infinium DNA methylation microarrays. Bioinformatics.

[58] Fortin J-P (2014). Functional normalization of 450k methylation array data improves replication in large cancer studies. Genome Biol..

[59] Triche TJ, Weisenberger DJ, Van Den Berg D, Laird PW, Siegmund KD (2013). Low-level processing of Illumina Infinium DNA Methylation BeadArrays. Nucleic Acids Res..

[60] Chen Y (2013). Discovery of cross-reactive probes and polymorphic CpGs in the Illumina Infinium HumanMethylation450 microarray. Epigenetics.

[61] Leek JT, Johnson WE, Parker HS, Jaffe AE, Storey JD (2012). The sva package for removing batch effects and other unwanted variation in high-throughput experiments. Bioinformatics.

[62] McGregor K (2016). An evaluation of methods correcting for cell-type heterogeneity in DNA methylation studies. Genome Biol..

[63] Ritchie ME (2015). limma powers differential expression analyses for RNA-sequencing and microarray studies. Nucleic Acids Res..

[64] Jaffe AE (2012). Bump hunting to identify differentially methylated regions in epigenetic epidemiology studies. Int. J. Epidemiol..

[65] Phipson B, Maksimovic J, Oshlack A (2016). missMethyl: An R package for analyzing data from Illumina’s HumanMethylation450 platform. Bioinformatics.

[66] Yousefi P (2015). Sex differences in DNA methylation assessed by 450 K BeadChip in newborns. BMC Genom..

[67] Suderman, M. *et al.* Sex-associated autosomal DNA methylation differences are wide-spread and stable throughout childhood (2017).

[68] Price ME (2013). Additional annotation enhances potential for biologically-relevant analysis of the Illumina Infinium HumanMethylation450 BeadChip array. Epigenetics Chromatin.

[69] Inoshita M (2015). Sex differences of leukocytes DNA methylation adjusted for estimated cellular proportions. Biol. Sex Differ..

[70] Singmann P (2015). Characterization of whole-genome autosomal differences of DNA methylation between men and women. Epigenetics Chromatin.

[71] Xu H (2014). Sex-biased methylome and transcriptome in human prefrontal cortex. Hum. Mol. Genet..

[72] Xia Y (2019). Sex-differential DNA methylation and associated regulation networks in human brain implicated in the sex-biased risks of psychiatric disorders. Mol. Psychiatry.

[73] Hall E (2014). Sex differences in the genome-wide DNA methylation pattern and impact on gene expression, microRNA levels and insulin secretion in human pancreatic islets. Genome Biol..

[74] Spiers H (2015). Methylomic trajectories across human fetal brain development. Genome Res..

