# Supplementary material for: Large-scale placenta DNA methylation integrated analysis reveals fetal sex-specific differentially methylated CpG sites and regions
Source: Sci Rep. 2022 Jun 7;12:9396. doi: 10.1038/s41598-022-13544-z (PMC9174475; doi:10.1038/s41598-022-13544-z)
Supplement: Supplementary file 13 — Supplementary Table and Figures. [file 41598_2022_13544_MOESM13_ESM.pdf]

Supplementary Table 1: Datasets included in this study. Included Full Term Analysis: Y/N variable indicating if samples from dataset were used in full term analysis. Inclusion Criteria Full Term Analysis: criteria to select samples for full term analysis. Included Early Term Analysis: Y/N variable indicating if samples from dataset were used in any of the trimester specific analyses. Inclusion Criteria Early Term Analysis: criteria to select samples for inclusion in trimester specific cohorts. N_Term/1stTerm/2ndTerm/3rdTerm: Number of samples (after duplicate removal, pre QC) included from dataset in each analysis subgroup. T = total count, M = male sex, F = female sex.

					**Full Term**			**Trimester 3**			**Trimester 2**			**Trimester 1**		
**GEOID**	**Included Full Term Analysis**	**Inclusion Criteria Full Term Analysis**	**Included Early Term Analysis**	**Inclusion Criteria Early Term Analysis**	T	M	F	T	M	F	T	M	F	**T**	M	F
GSE108567	Y	Via Gestational Age	Y	Via Gestational Age	45	23	22	12	6	6	0	0	0	0	0	0
GSE100197	Y	Term control	Y	Preterm control	7	3	4	21	14	7	2	1	1	0	0	0
GSE98224	Y	Term control, AGA	Y	Preterm, control, AGA	9	6	3	5	4	1	0	0	0	0	0	0
GSE106089	N	NA	Y	All	0	0	0	0	0	0	46	28	18	0	0	0
GSE103413	N	NA	Y	Chorionic villus	0	0	0	0	0	0	2	0	2	0	0	0
GSE98938	Y	chorion, trophoblast, villi	Y	chorion, villi, trophoblast	6	3	3	0	0	0	4	2	2	0	0	0
GSE93208	N	NA	Y	All	0	0	0	0	0	0	0	0	0	19	10	9
GSE71719	Y	Bisulfite converted	N	NA	23	12	11	0	0	0	0	0	0	0	0	0
GSE71678	Y	All	N	NA	322	171	151	0	0	0	0	0	0	0	0	0
GSE75248	Y	AGA samples	N	NA	174	90	84	0	0	0	0	0	0	0	0	0
GSE75196	Y	Healthy	N	NA	16	7	9	0	0	0	0	0	0	0	0	0
GSE69502	N	NA	Y	Chorionic Villi, Controls	0	0	0	0	0	0	16	0	16	0	0	0
GSE74738	Y	Controls	N	NA	2	1	1	0	0	0	0	0	0	0	0	0
GSE66210	N	NA	N	Chorionic villus, normal	0	0	0	0	0	0	0	0	0	12	6	6
				**SUM**	604	316	288	38	24	14	70	31	39	31	16	15

Supplementary Table 2: Differential gene expression results for genes closest to significant (fwerArea < 0.1) DMRs from full term analysis. Data from GSE75010. Output is from limma topTable() function. A significance threshold of .05 was applied to Benjamini-Hochberg–corrected p–values.

gene	logFC	p-value	Adjusted p-value
C5orf63	0.0272	0.1562	0.4687
CDKN1C	-0.003	0.532	0.7557
CROT	0.0002	0.9918	0.9918
ERCC6L2	0.024	0.0955	0.408
ERCC6L2	0.013	0.2208	0.4839
ERCC6L2	-0.006	0.6543	0.7557
HOXA4	0.0113	0.242	0.4839
SNCA	-0.028	0.102	0.408
ZBED9	0.0035	0.6927	0.7557
ZNF175	0.0105	0.6515	0.7557
ZNF175	0.0051	0.6811	0.7557
ZNF300	-0.049	0.0022	**0.0263**

Supplementary Figures

Supplementary Figure 1: PCA plot of full term placenta samples before and after batch effect correction. Top triangle shows principal components generated from methylation M-values at all autosomal probes passing QC prior to quantile normalization or surrogate variable analysis. Bottom triangle shows residuals of quantile normalized autosomal M-values regressed onto estimated surrogate variables.

Supplementary Figure 2: PCA plot of 3rd Trimester placenta samples before and after batch effect correction. Top triangle shows principal components generated from methylation M-values at all autosomal probes passing QC prior to quantile normalization or surrogate variable analysis. Bottom triangle shows residuals of quantile normalized autosomal M-values regressed onto estimated surrogate variables.

Supplementary Figure 3: PCA plot of 2nd Trimester placenta samples before and after batch effect correction. Top triangle shows principal components generated from methylation M-values at all autosomal probes passing QC prior to quantile normalization or surrogate variable analysis. Bottom triangle shows residuals of quantile normalized autosomal M-values regressed onto estimated surrogate variables.

Supplementary Figure 4: PCA plot of 1st Trimester placenta samples before and after batch effect correction. Top triangle shows principal components generated from methylation M-values at all autosomal probes passing QC prior to quantile normalization or surrogate variable analysis. Bottom triangle shows residuals of quantile normalized autosomal M-values regressed onto estimated surrogate variables.

Supplementary Figure 5: Exploration of fetal sex differences in placenta DNAm in different genomics regions. a Density plots of % DNAm values in regions defined by proximity to CpG islands **b** Density plots of % DNAm values in regions defined by proximity to annotated regulatory features defined by ENCODE per the Illumina 450K manifest **c** Distribution of probes mapping to regions defined by proximity to CpG islands for all probes analyzed in full term analysis and probes found to be differentially methylated (n = 5,212) in same analysis. P-value represents chi-square test evaluating significance of counts for these two groups of probes. **d** Distribution of probes mapping to regions defined by annotated regulatory features for all probes analyzed in full term analysis and probes found to be differentially methylated (n = 5,212) in same analysis. P-value represents chi-square test evaluating significance of counts for these two groups of probes.

Supplementary Figure 6: Additional explorations of mean difference in full term analysis. a Volcano Plot **b-c** Study-specific effects of individual CpG sites showing highest degree of hypermethylation in males (cg01382982; **b**) and females (cg22905511; **c**) in the full dataset, in the full term analysis.

Supplementary Figure 7: Distribution of absolute value of mean differences in sites that do discriminate placenta cell types (gray, n = 1,285) and sites that do not (orange, n = 3927), among the genome-wide significant sites from the full term analysis. Cell type discriminating probes determined by Yuan et al.

Supplementary Figure 8: Manhattan plots showing single site association results for each early term analysis. In each plot, top half restricted to sites hypermethylated in males, bottom half restricted to sites hypermethylated in females. Orange line represents threshold of 1E-8, the considered cutoff for genome-wide significance.

Supplementary Figure 9: Volcano plot for full term DMR analysis. The x-axis measures the average methylation difference between male and female fetuses, and the y-axis measures the size of the DMR in base pairs. The blue points represent DMRs considered significant (fwerArea < 0.1)

Supplementary Figures 10 - 17: Additional plots depicting significant DMRs from full term analysis across all gestational periods. Remaining DMRs exceeding fwerArea threshold of 0.1 as seen in Table 4 (excluding top-ranked DMR in ZNF175 promoter, see Figure 4). In each figure, DMRs are plotted as percent methylation as a function of genomic position. Dots indicate samples and solid lines indicate smooth lines through male sample values (blue) and female sample values (gold). Actual identified DMR region indicated via dashed lines. **a** Full Term Samples. **b** 1st Trimester Samples **c** 2nd Trimester Samples **d** 3rd Trimester Samples.

Supplementary Figures 18 - 21: Exploration of cell type heterogeneity in additional DMRs. Remaining DMRs containing cell type discriminating probes as determined by Yuan et al (see Table 4). DMRs are plotted as percent methylation as a function of genomic position. Dots indicate samples and solid lines indicate smooth lines through male sample values (blue) and female sample values (gold). Actual identified DMR region indicated via dashed lines. **a** DMR identified in full term analysis, plotted using full term samples from present study. **b - d** The same region, but plotted using samples from different datasets. **b** Term samples and individual placenta cell types from Yuan et al. **c** 1st Trimester samples from present study. **d** 1st trimester samples and individual placenta cell types from Yuan et al. In **b** + **d**, fractions indicate the proportion of probes in the region that were annotated by Yuan et al. as distinguishing that cell type in full term and 1st trimester samples, respectively. Villi samples were not evaluated in this manner by Yuan et al. as this is the unsorted bulk tissue.

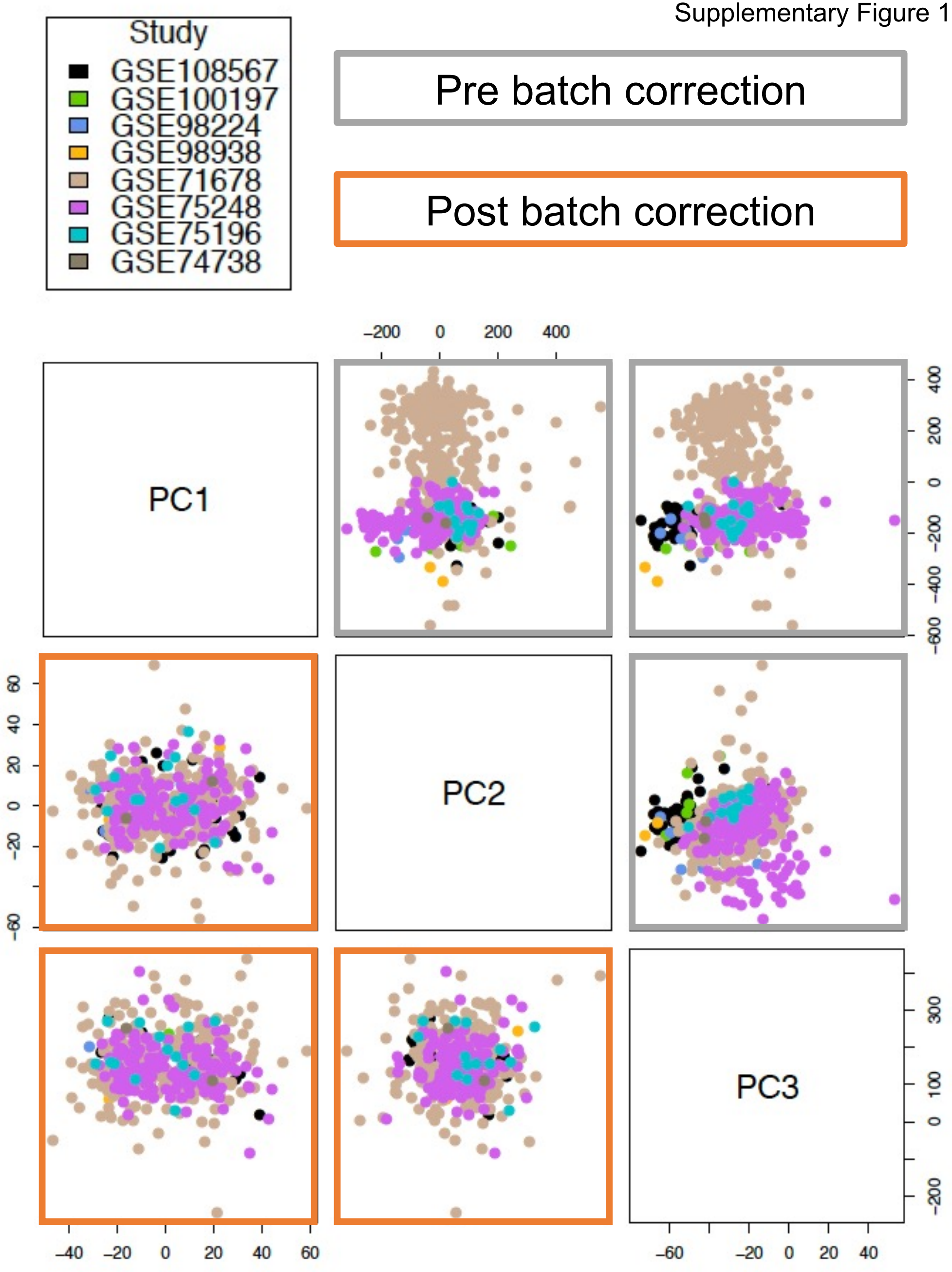
Study
GSE108567
GSE100197
GSE98224
GSE98938
GSE71678
GSE75248
GSE75196
GSE74738
Pre batch correction
Post batch correction
PC1
PC2
PC3

Supplementary Figure 2

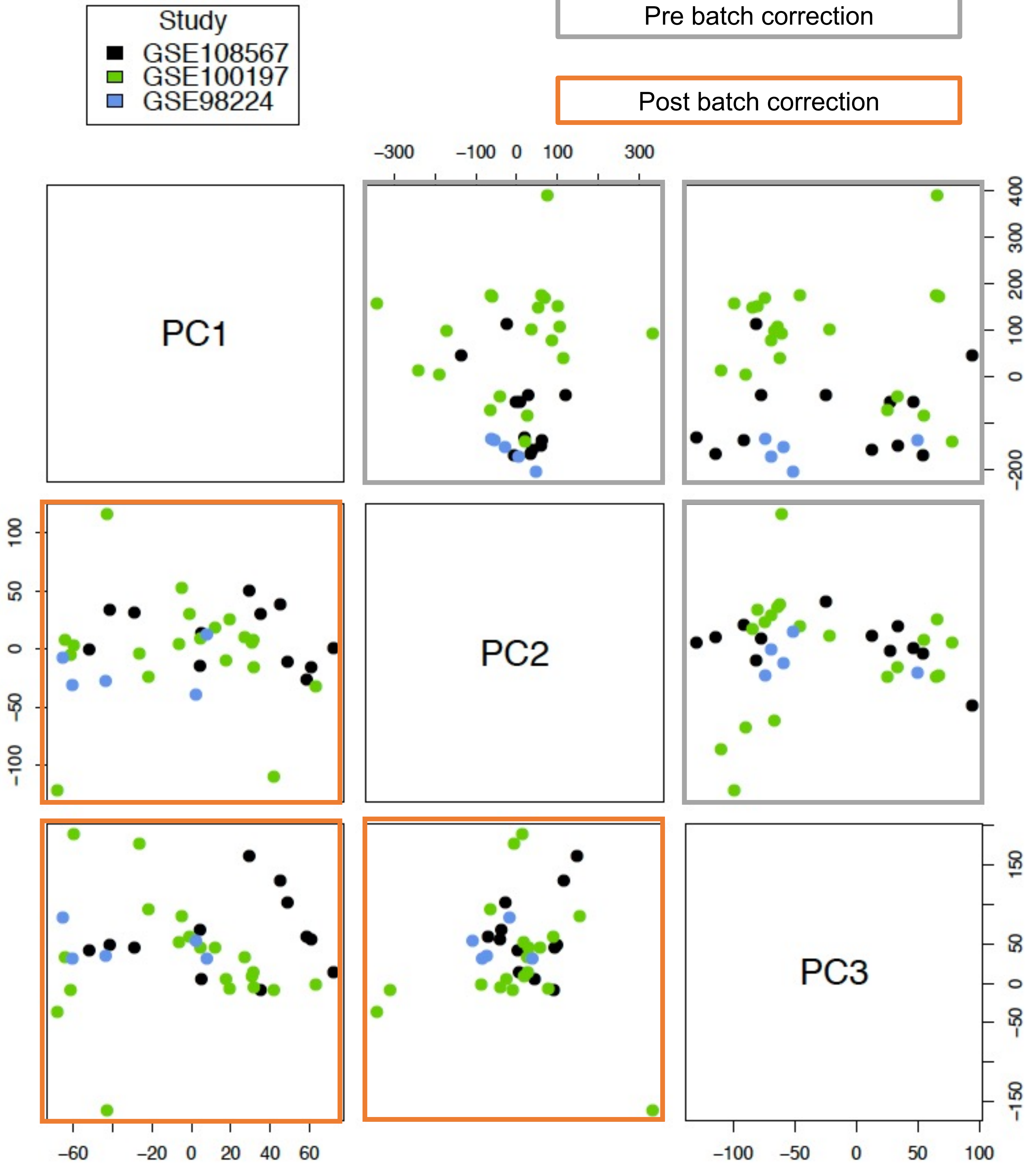

Supplementary Figure 3

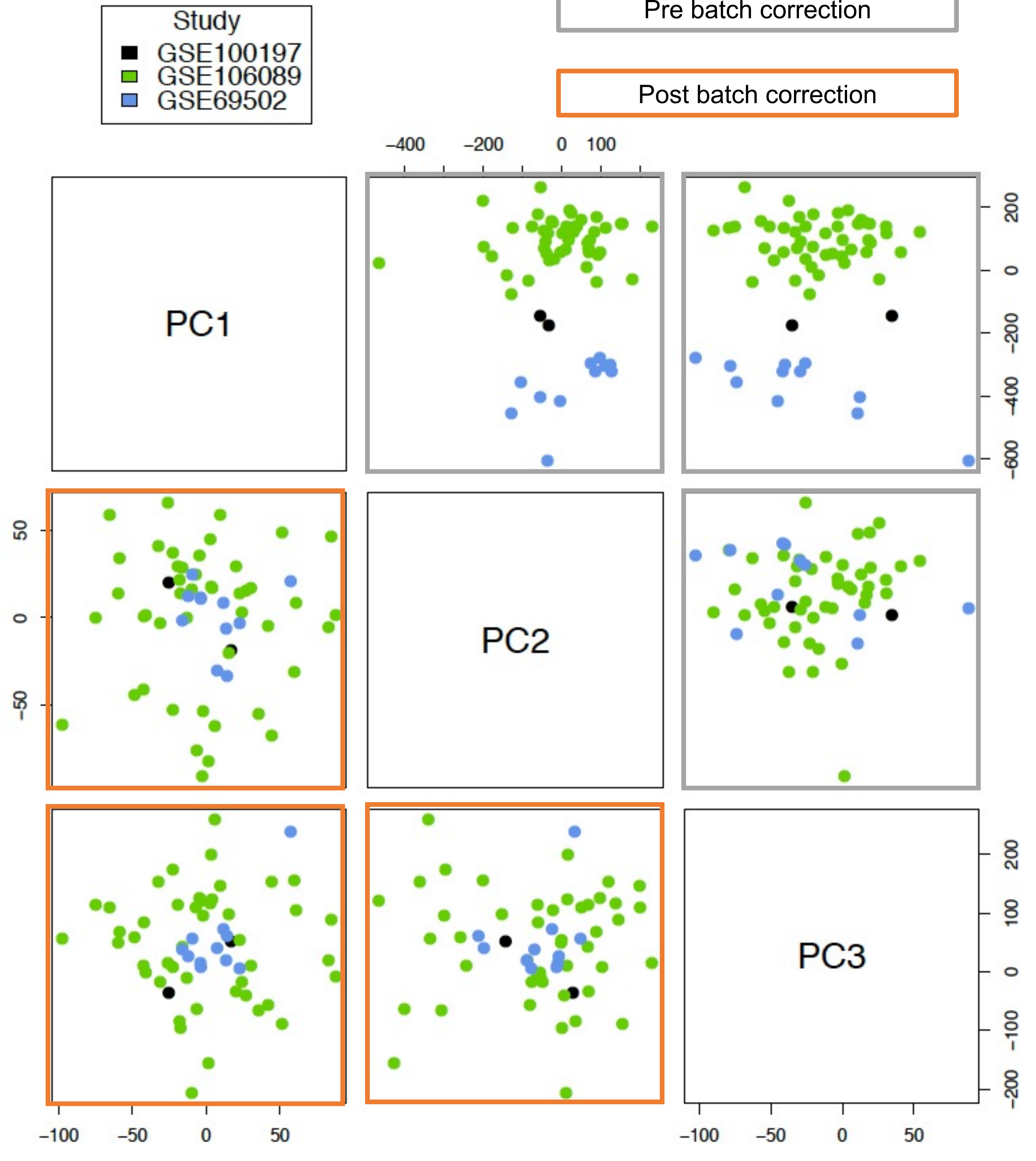

Supplementary Figure 4

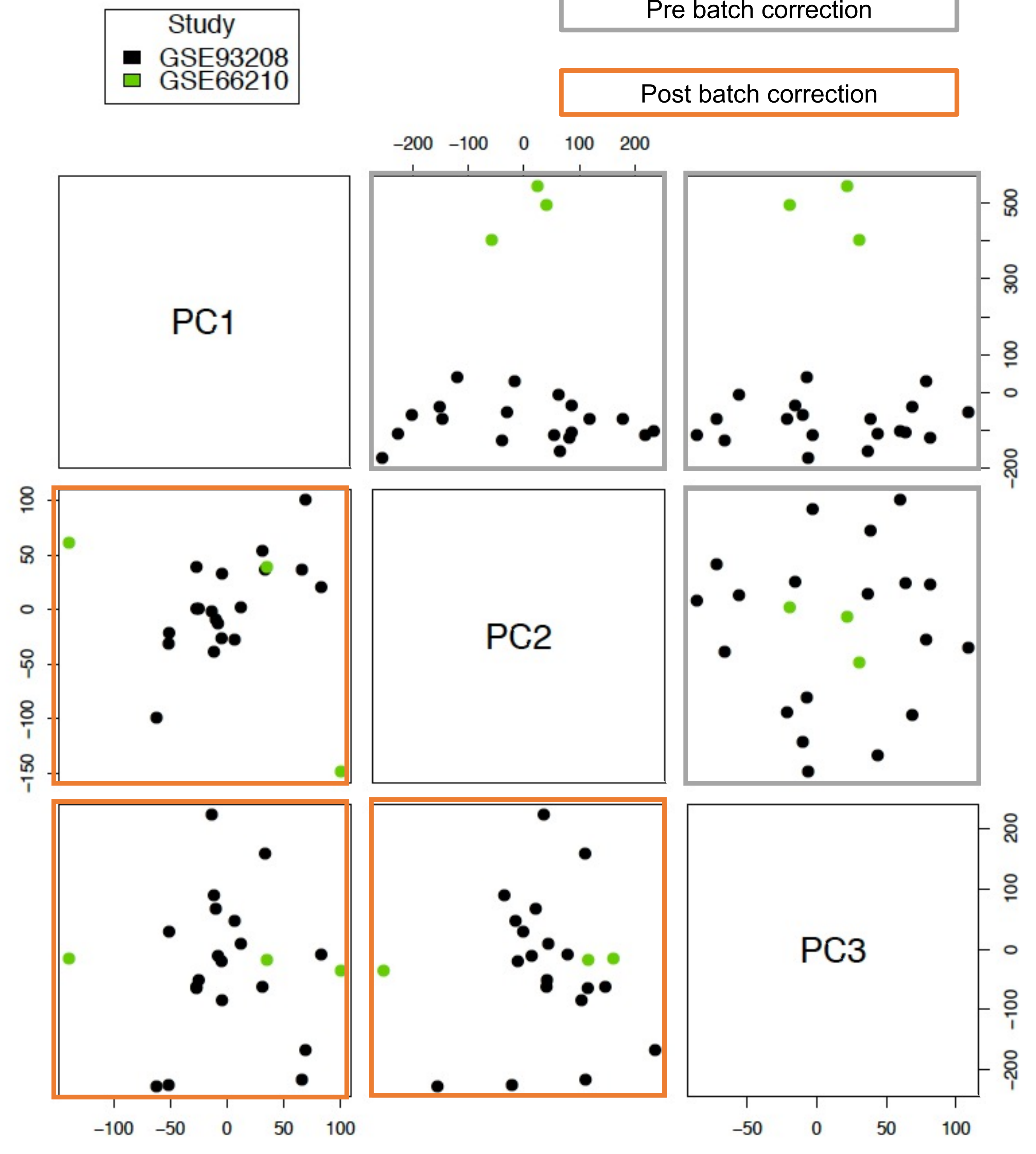

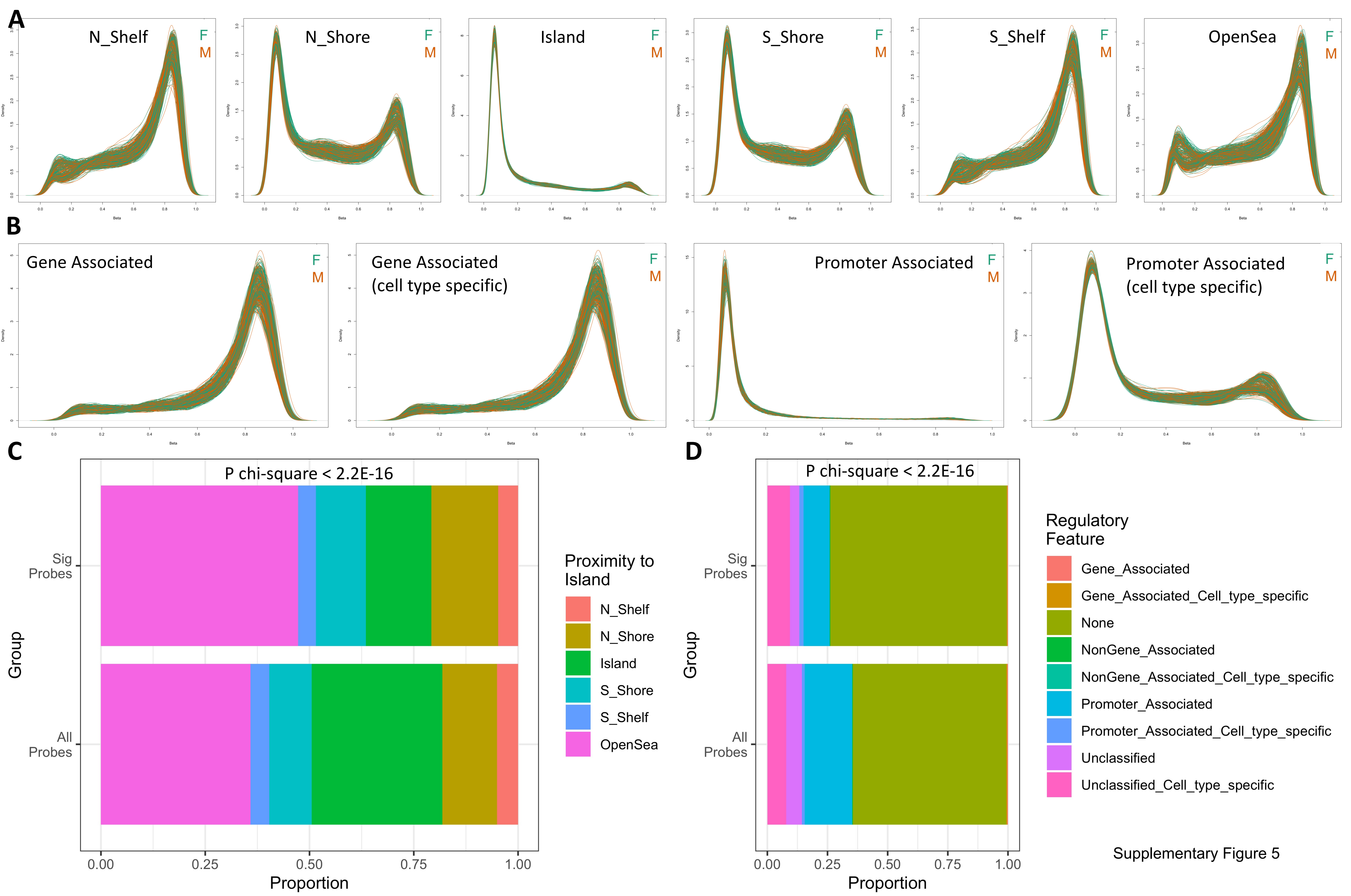

Supplementary Figure 5

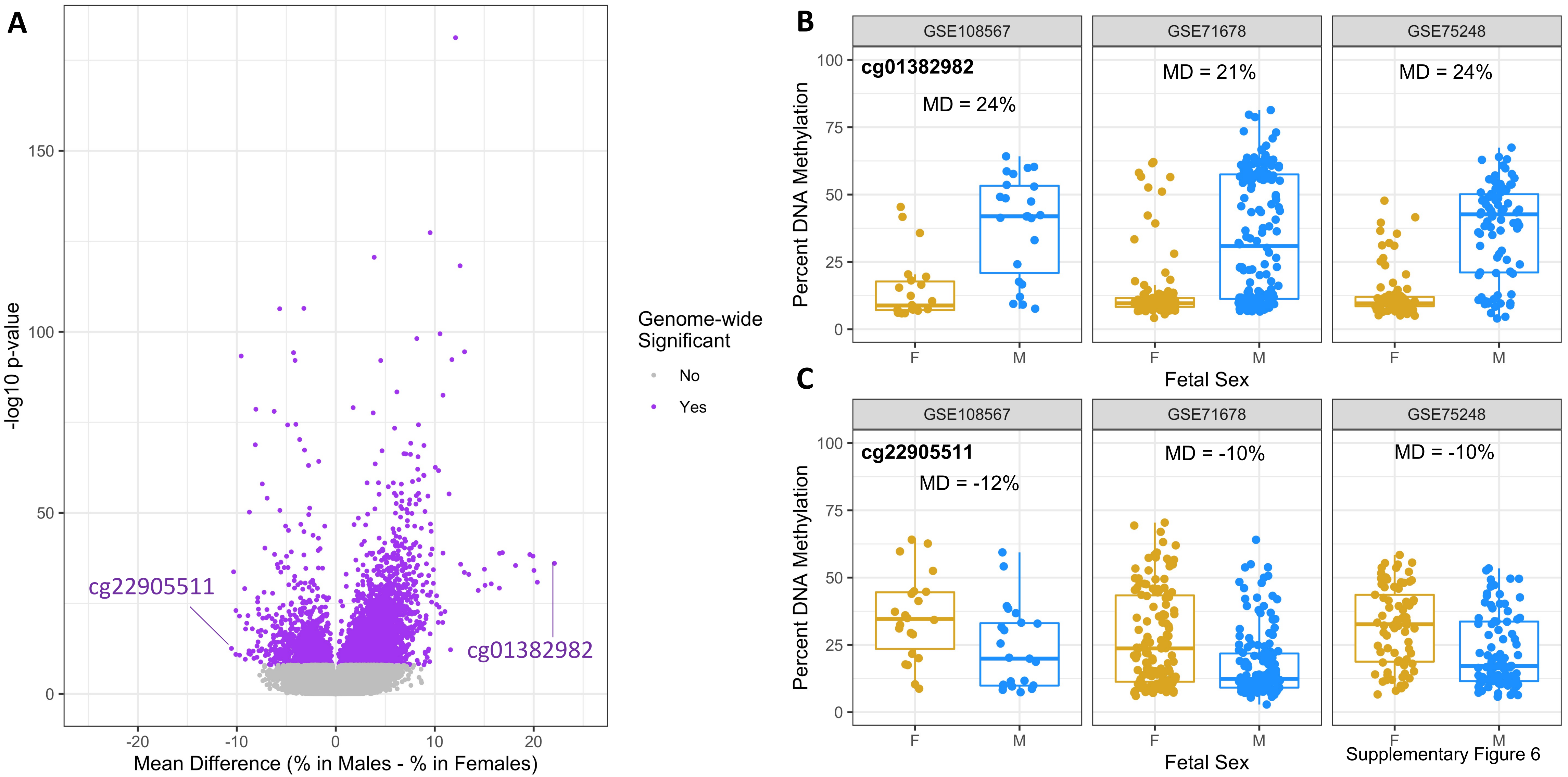

Supplementary Figure 6

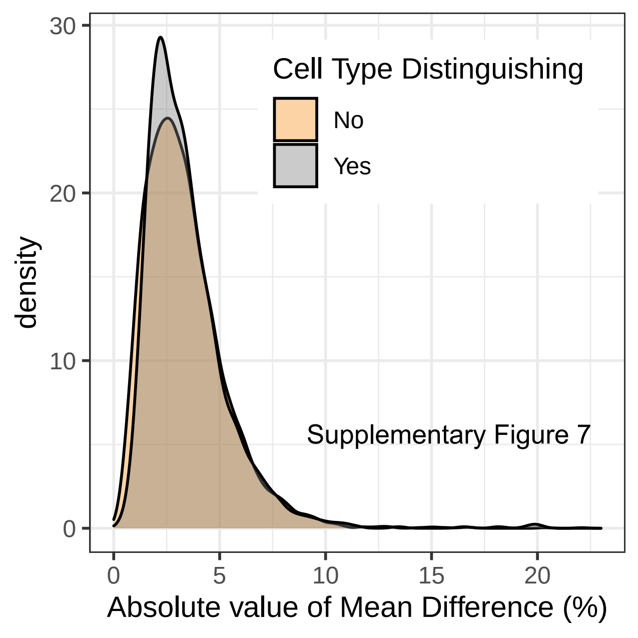

Cell Type Distinguishing
No
Yes
Supplementary Figure 7
density
Absolute value of Mean Difference (%)
0
10
20
30
0
5
10
15
20

Supplementary Figure 8

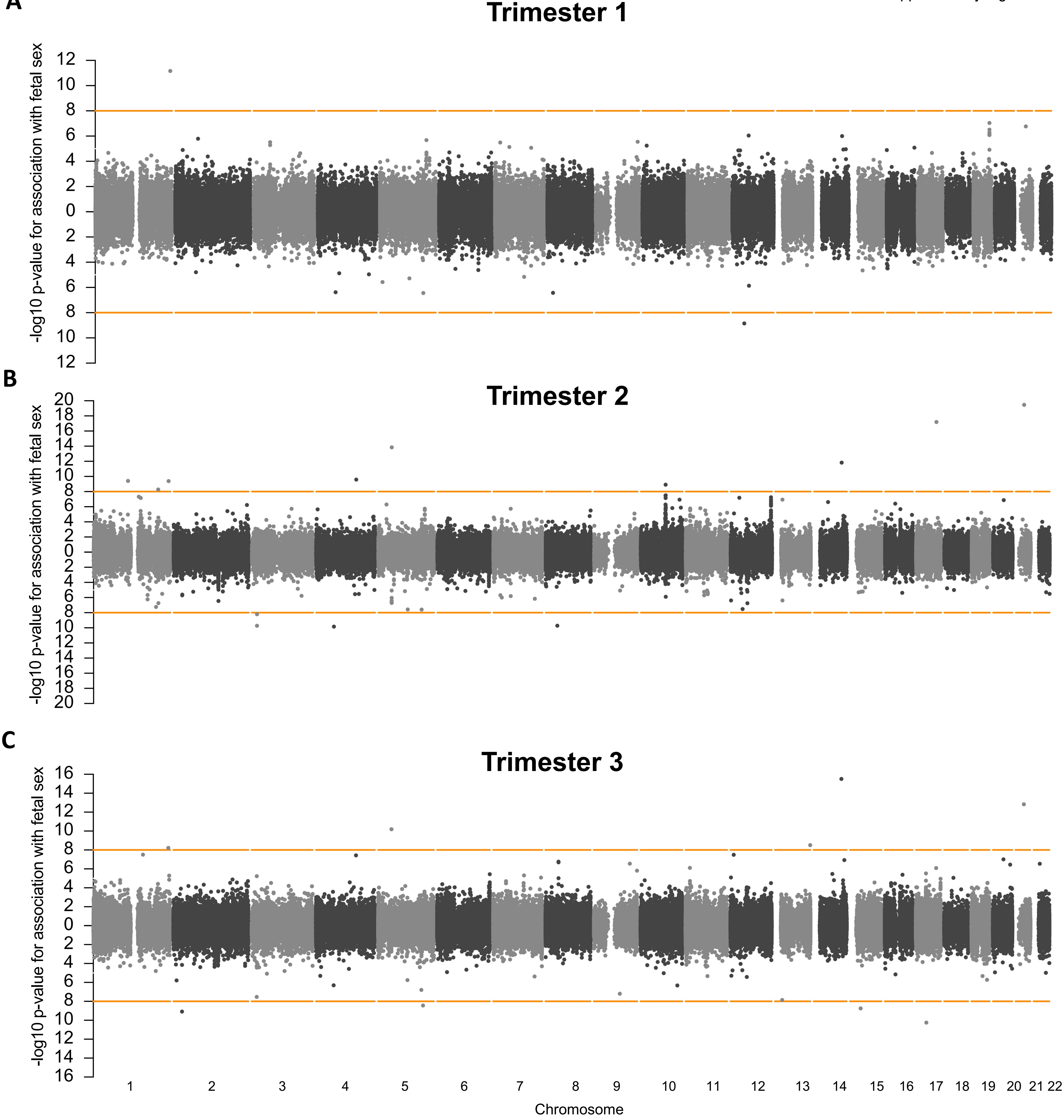

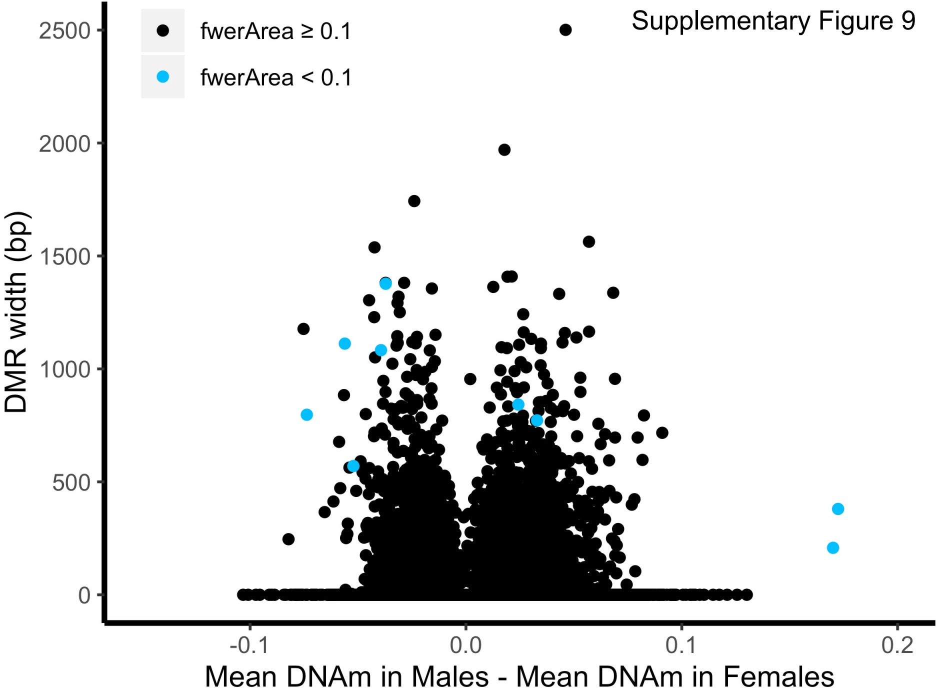
Supplementary Figure 9
fwerArea ≥ 0.1
fwerArea < 0.1
DMR width (bp)
0
500
1000
1500
2000
2500
-0.1
0.0
0.1
0.2
Mean DNAm in Males - Mean DNAm in Females

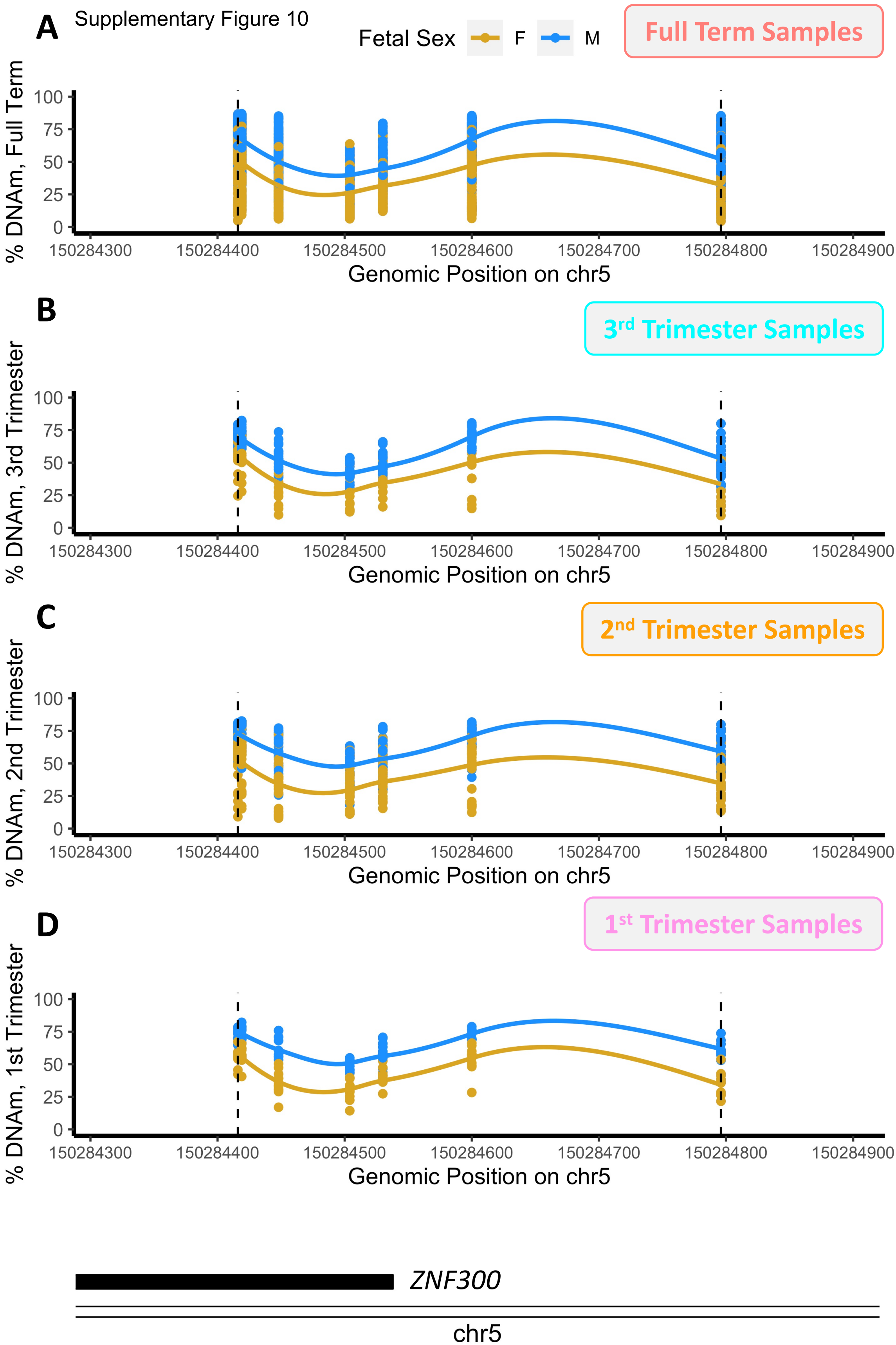
A
Supplementary Figure 10
Fetal Sex
F
M
Full Term Samples
% DNAm, Full Term
100
75
50
25
0
150284300
150284400
150284500
150284600
150284700
150284800
150284900
Genomic Position on chr5
B
3rd Trimester Samples
% DNAm, 3rd Trimester
Genomic Position on chr5
C
2nd Trimester Samples
% DNAm, 2nd Trimester
Genomic Position on chr5
D
1st Trimester Samples
% DNAm, 1st Trimester
Genomic Position on chr5
ZNF300
chr5

Supplementary Figure 11

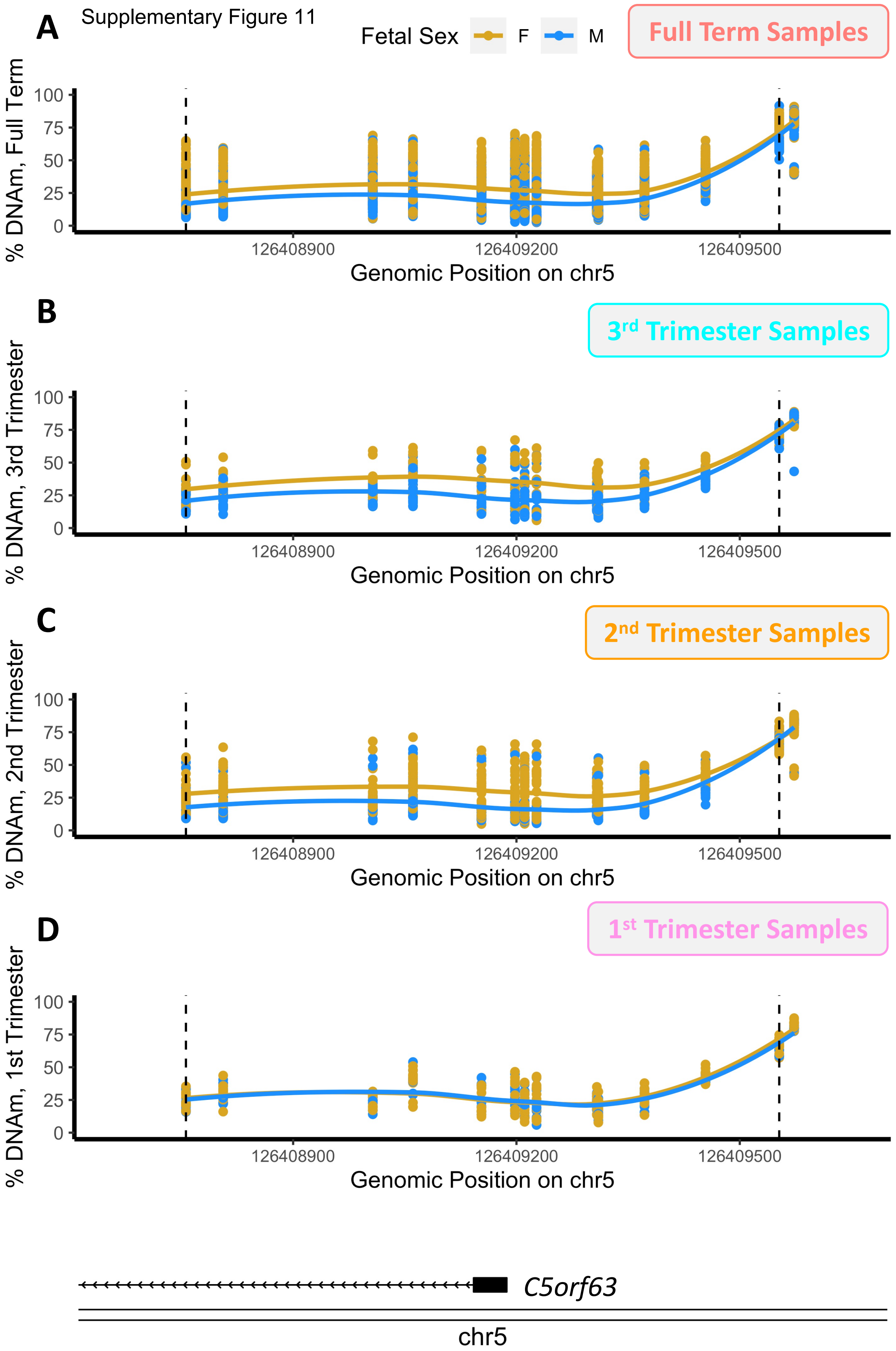

Supplementary Figure 12

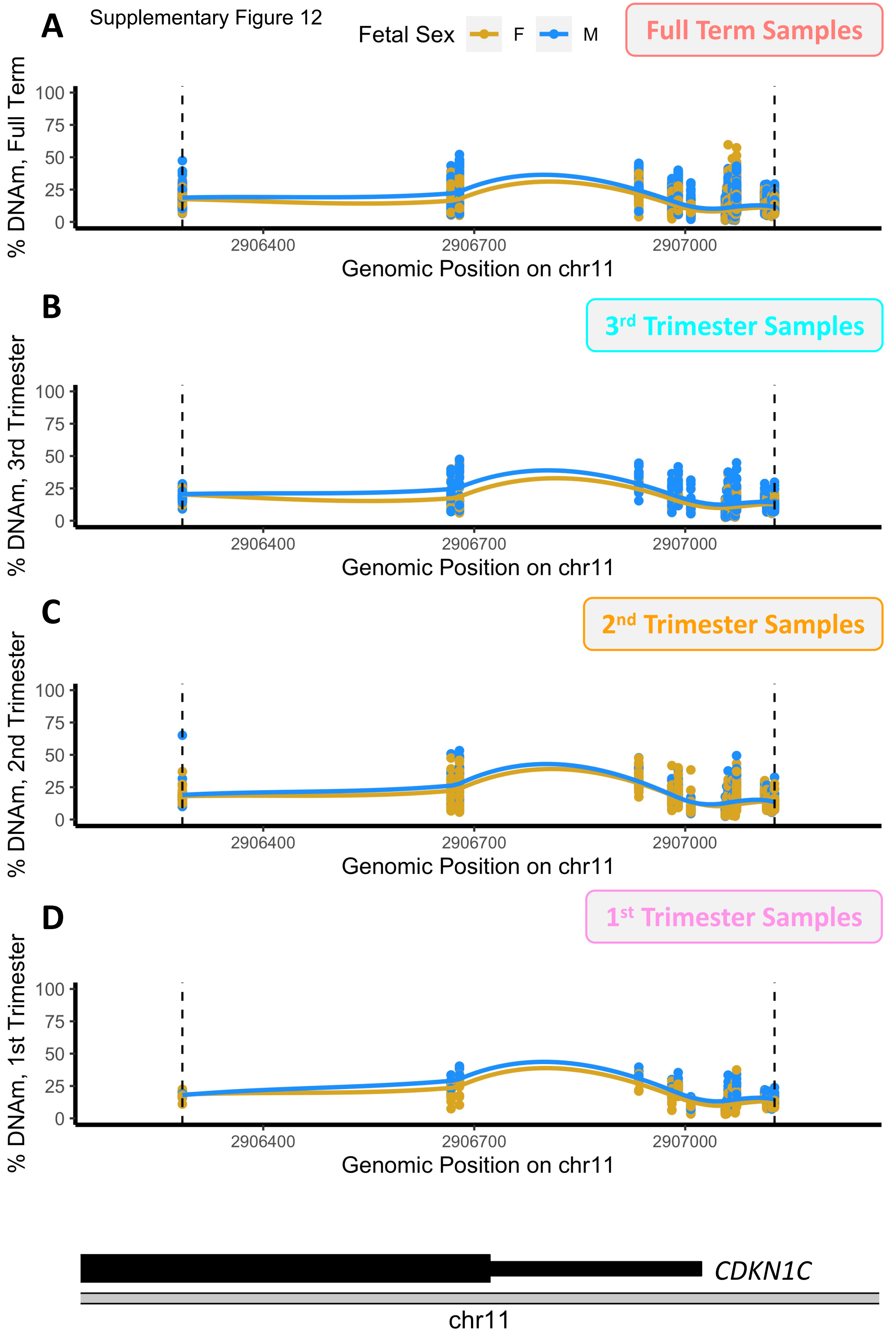

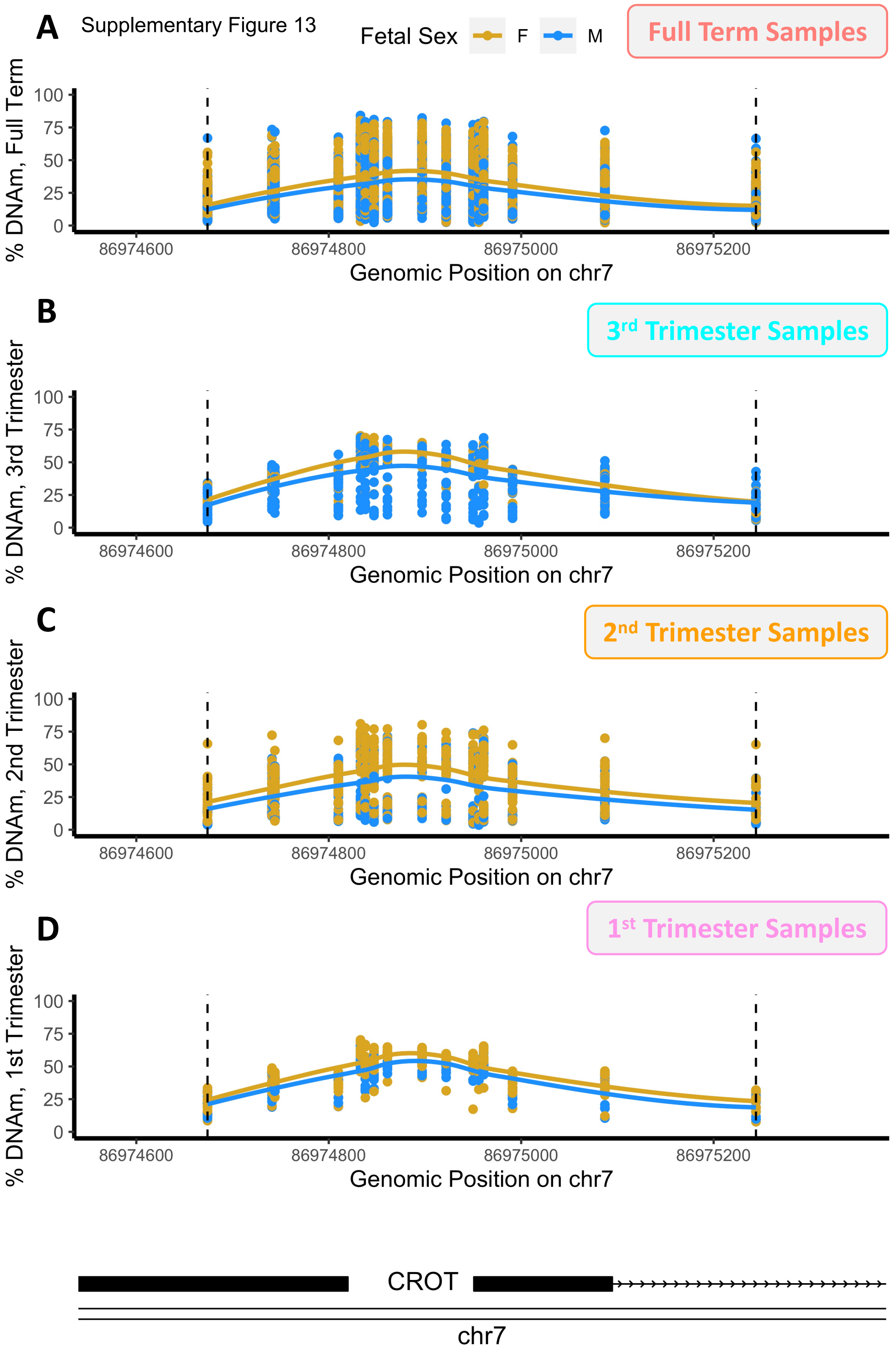
A
Supplementary Figure 13
Fetal Sex
F
M
Full Term Samples
% DNAm, Full Term
100
75
50
25
0
86974600
86974800
86975000
86975200
Genomic Position on chr7
B
3rd Trimester Samples
% DNAm, 3rd Trimester
Genomic Position on chr7
C
2nd Trimester Samples
% DNAm, 2nd Trimester
Genomic Position on chr7
D
1st Trimester Samples
% DNAm, 1st Trimester
Genomic Position on chr7
CROT
chr7

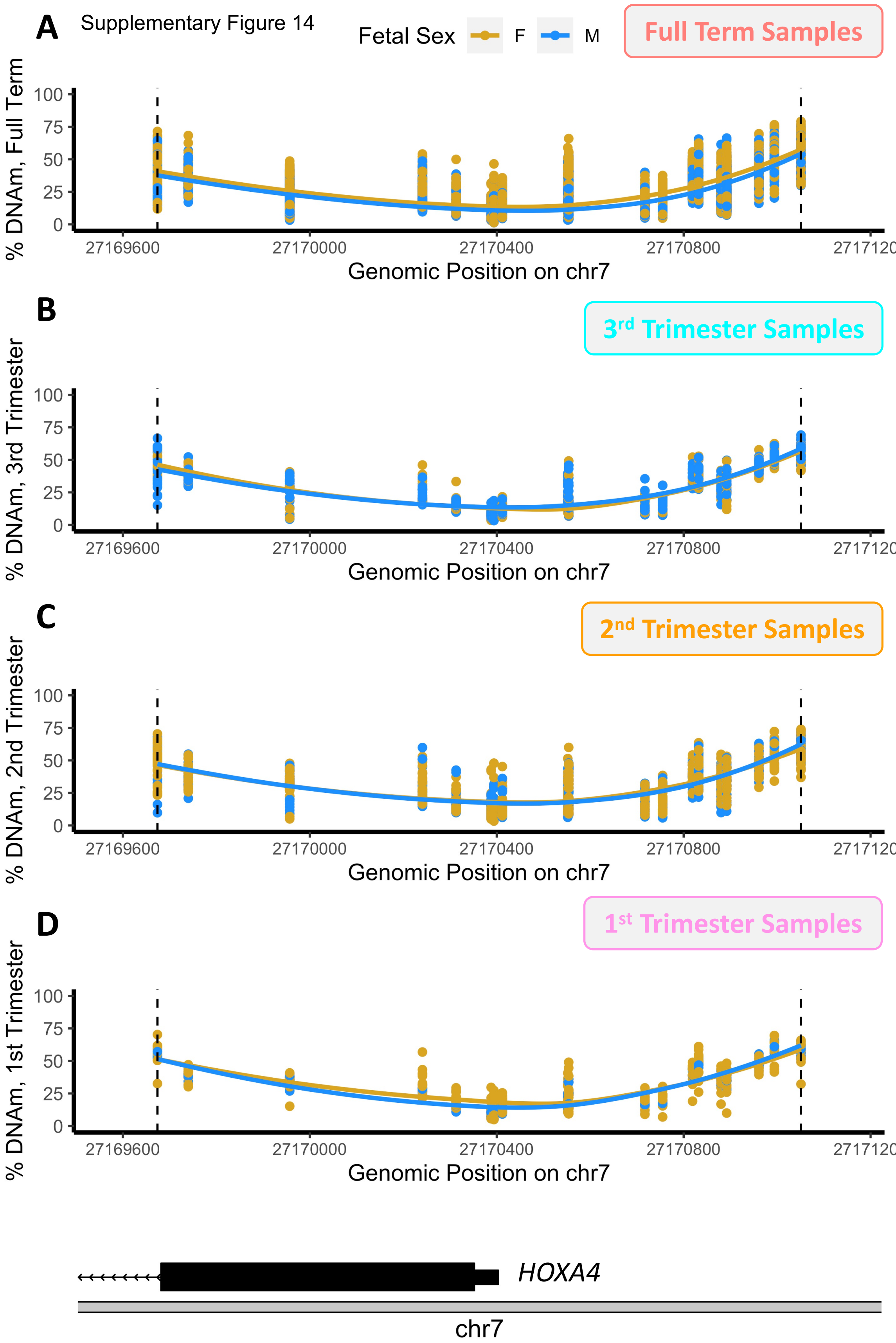
A
Supplementary Figure 14
Fetal Sex
F
M
Full Term Samples
% DNAm, Full Term
100
75
50
25
0
27169600
27170000
27170400
27170800
2717120
Genomic Position on chr7
B
3rd Trimester Samples
% DNAm, 3rd Trimester
C
2nd Trimester Samples
% DNAm, 2nd Trimester
D
1st Trimester Samples
% DNAm, 1st Trimester
HOXA4
chr7

Supplementary Figure 15

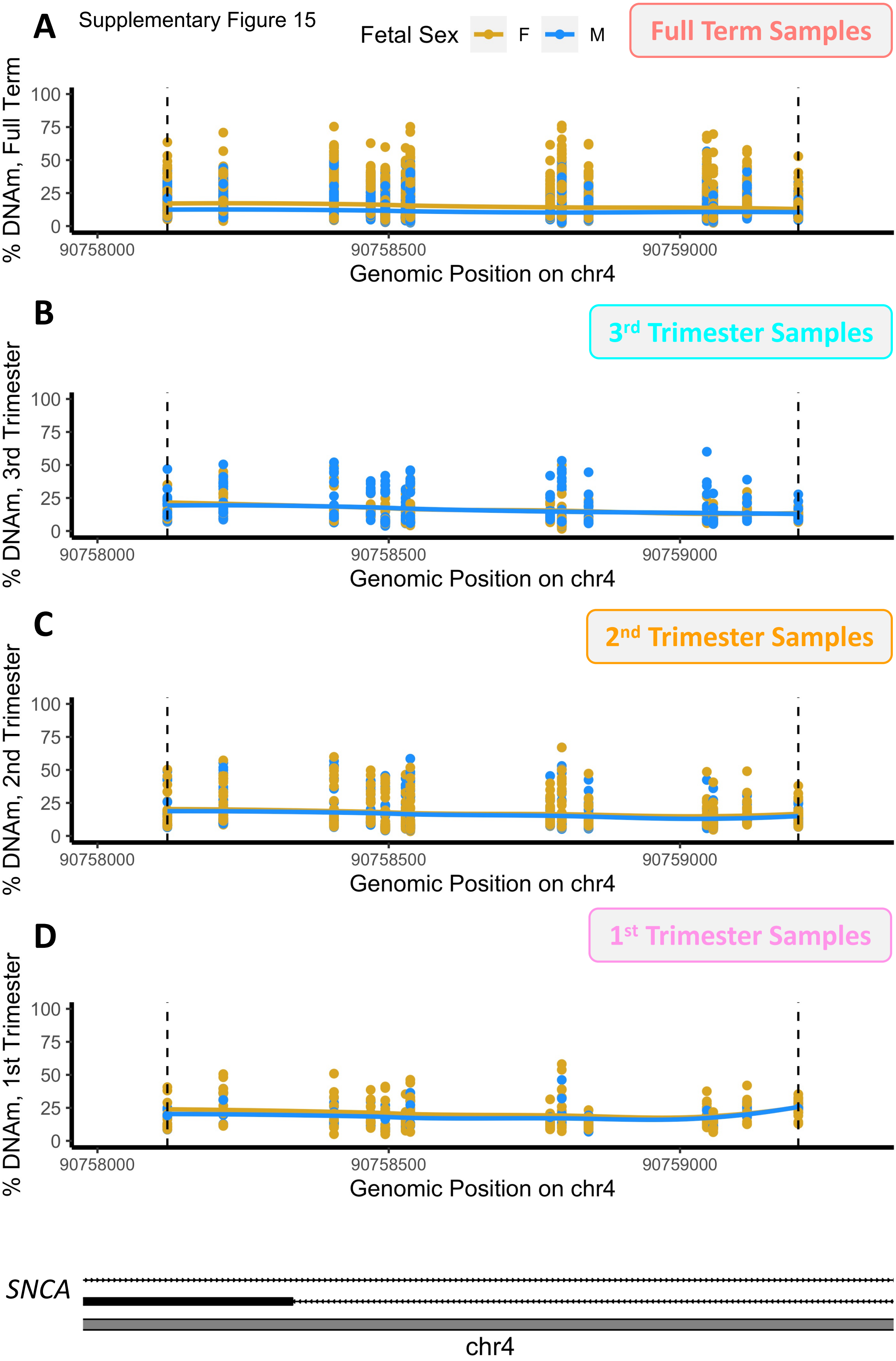

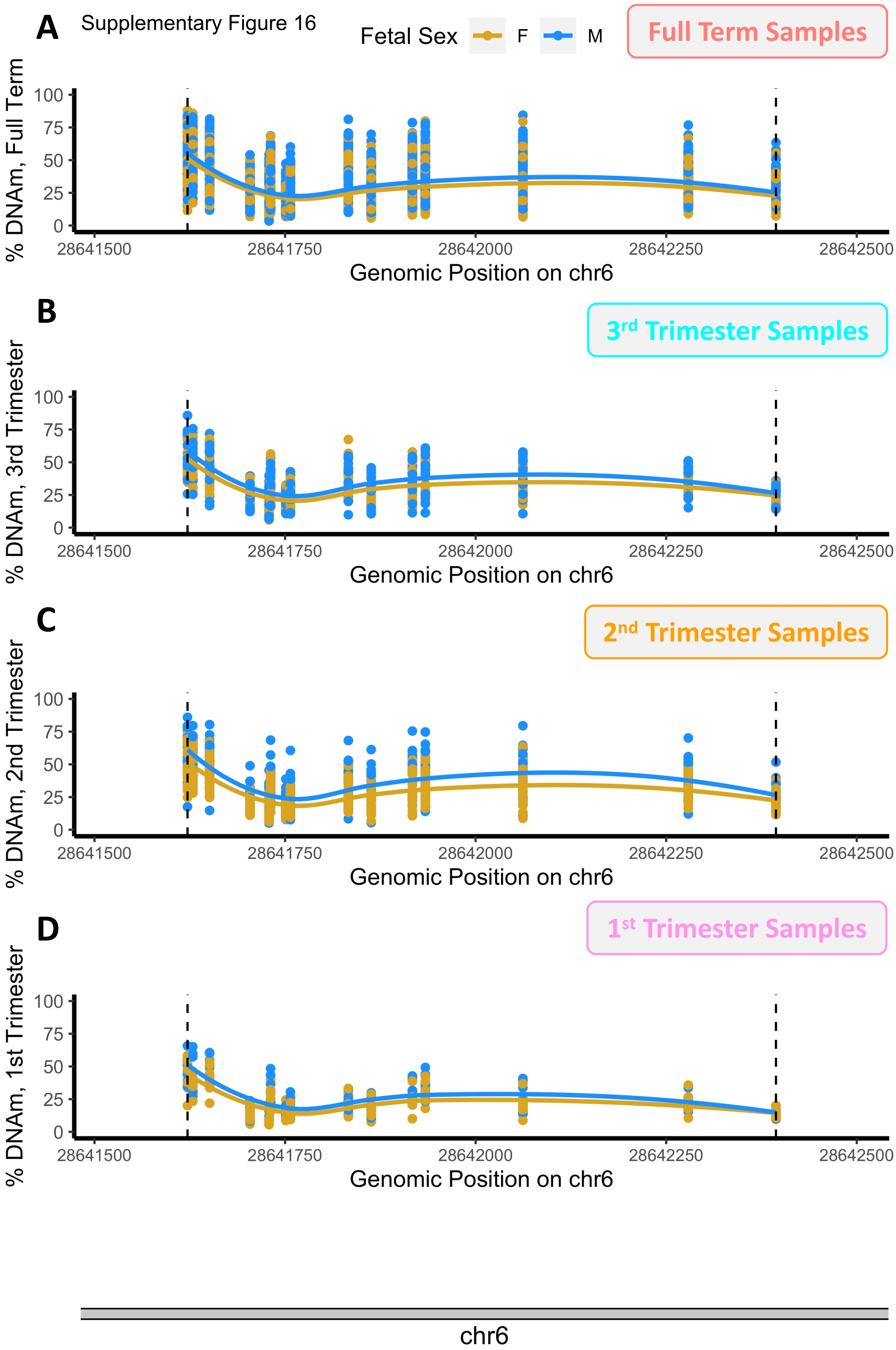

A
Supplementary Figure 16
Fetal Sex
F
M
Full Term Samples
% DNAm, Full Term
100
75
50
25
0
28641500
28641750
28642000
28642250
28642500
Genomic Position on chr6
B
3rd Trimester Samples
% DNAm, 3rd Trimester
Genomic Position on chr6
C
2nd Trimester Samples
% DNAm, 2nd Trimester
Genomic Position on chr6
D
1st Trimester Samples
% DNAm, 1st Trimester
Genomic Position on chr6
chr6

Supplementary Figure 17

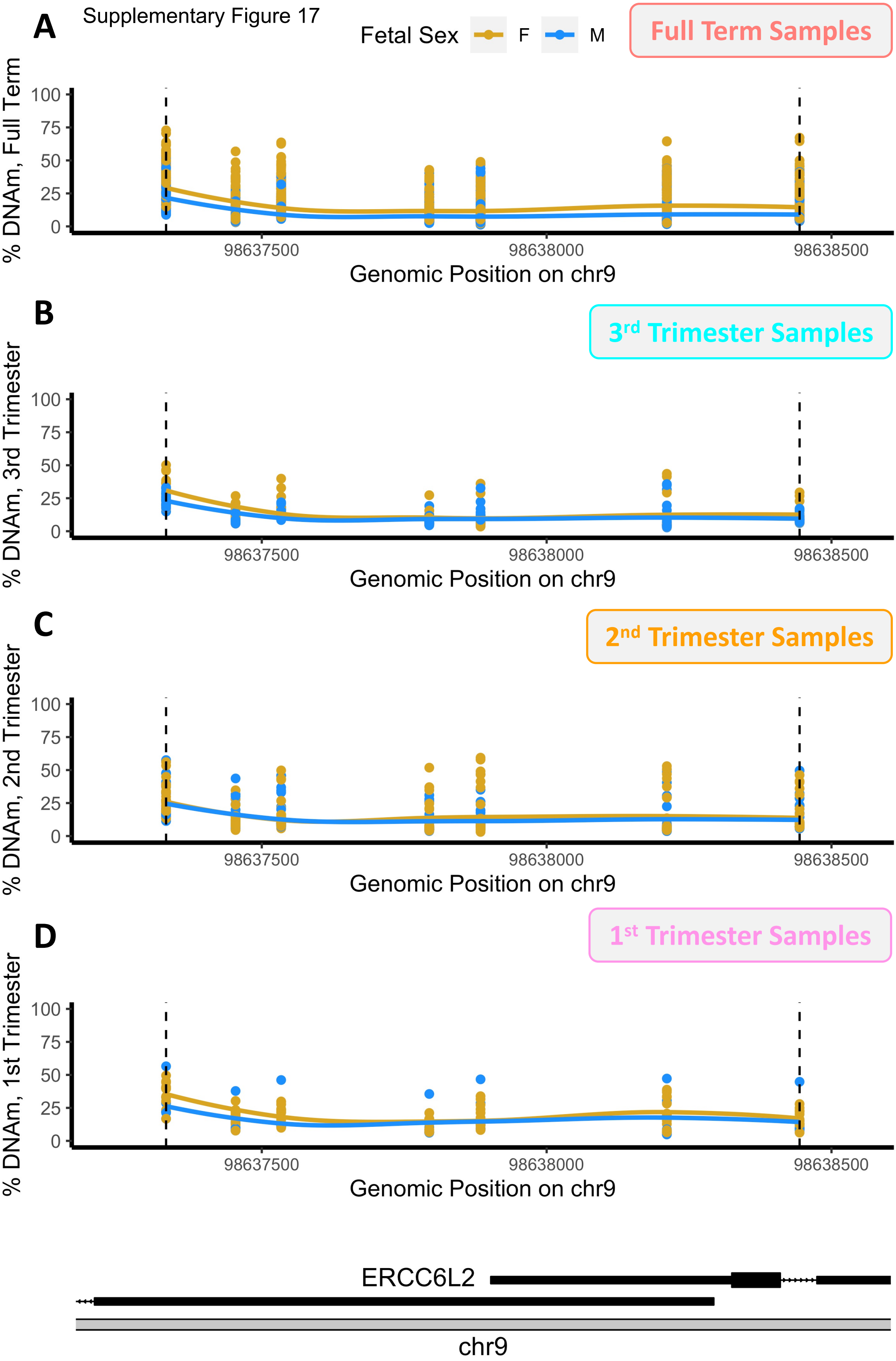

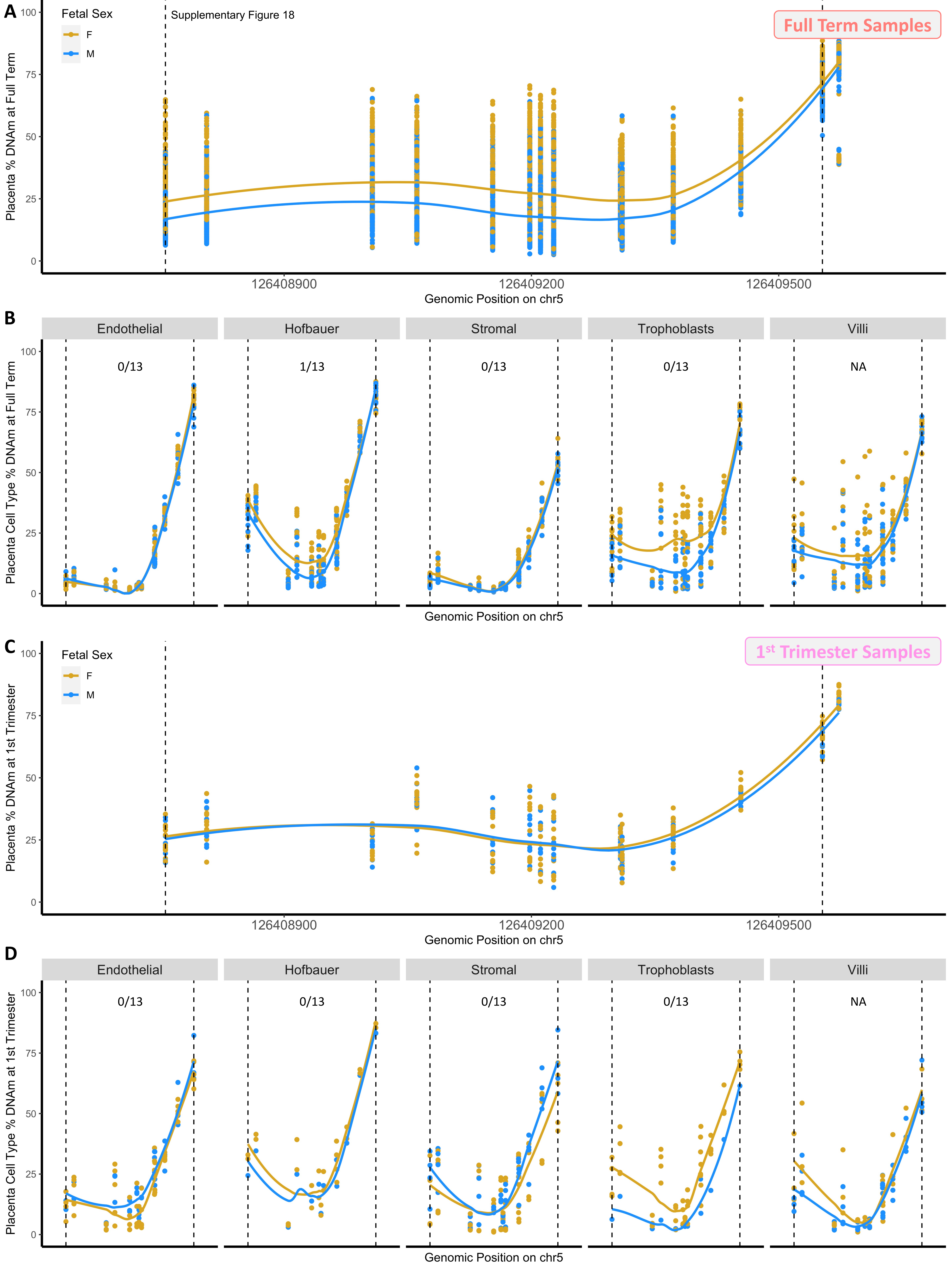

A
Supplementary Figure 18
Full Term Samples
Fetal Sex
F
M
Placenta % DNAm at Full Term
126408900
126409200
126409500
Genomic Position on chr5
B
Endothelial
Hofbauer
Stromal
Trophoblasts
Villi
0/13
1/13
0/13
0/13
NA
Placenta Cell Type % DNAm at Full Term
Genomic Position on chr5
C
1st Trimester Samples
Fetal Sex
F
M
Placenta % DNAm at 1st Trimester
126408900
126409200
126409500
Genomic Position on chr5
D
Endothelial
Hofbauer
Stromal
Trophoblasts
Villi
0/13
0/13
0/13
0/13
NA
Placenta Cell Type % DNAm at 1st Trimester
Genomic Position on chr5

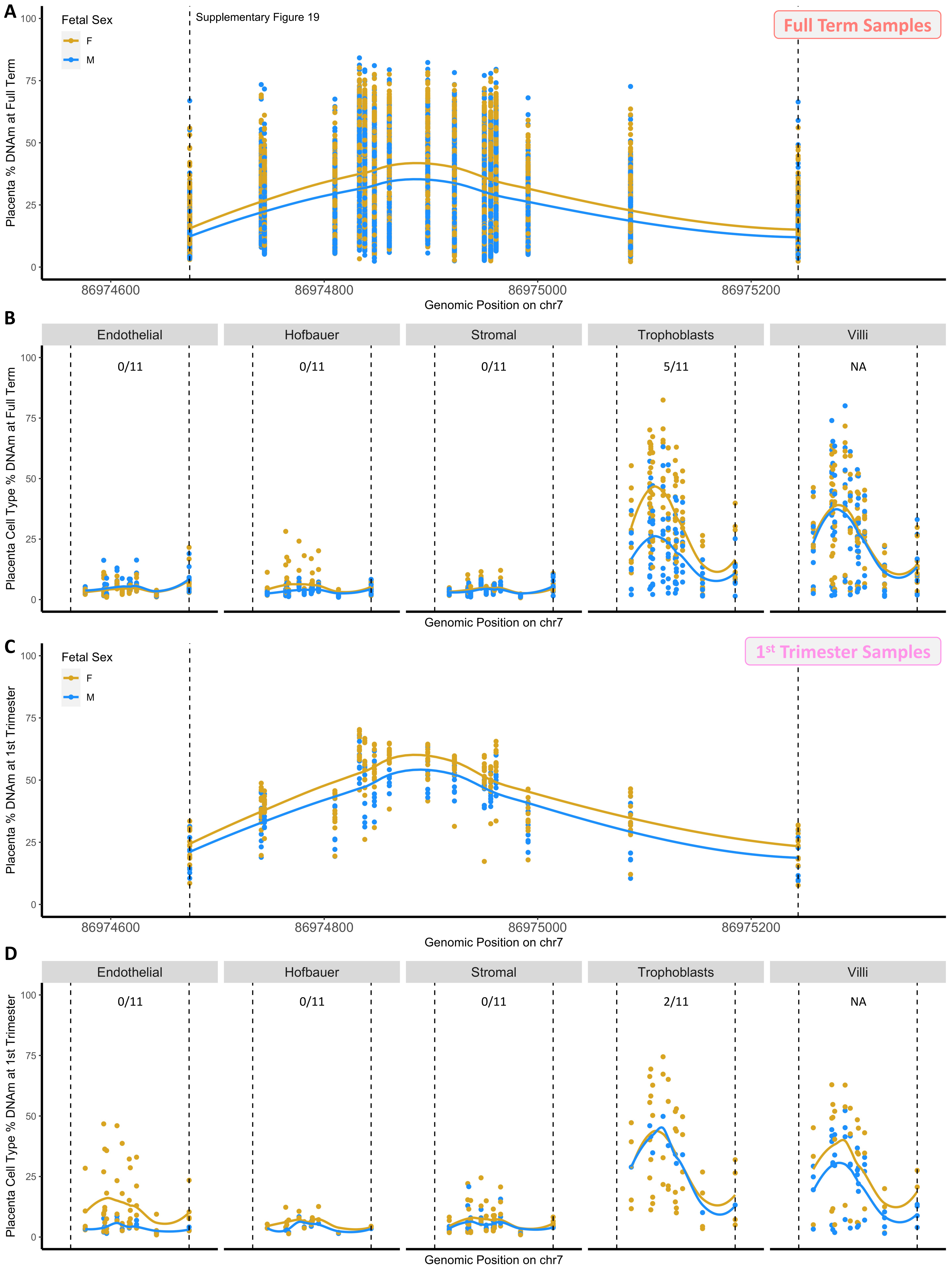
Supplementary Figure 19
A
Full Term Samples
Fetal Sex
F
M
Placenta % DNAm at Full Term
Genomic Position on chr7
B
Endothelial
Hofbauer
Stromal
Trophoblasts
Villi
0/11
0/11
0/11
5/11
NA
Placenta Cell Type % DNAm at Full Term
Genomic Position on chr7
C
1st Trimester Samples
Fetal Sex
F
M
Placenta % DNAm at 1st Trimester
Genomic Position on chr7
D
Endothelial
Hofbauer
Stromal
Trophoblasts
Villi
0/11
0/11
0/11
2/11
NA
Placenta Cell Type % DNAm at 1st Trimester
Genomic Position on chr7

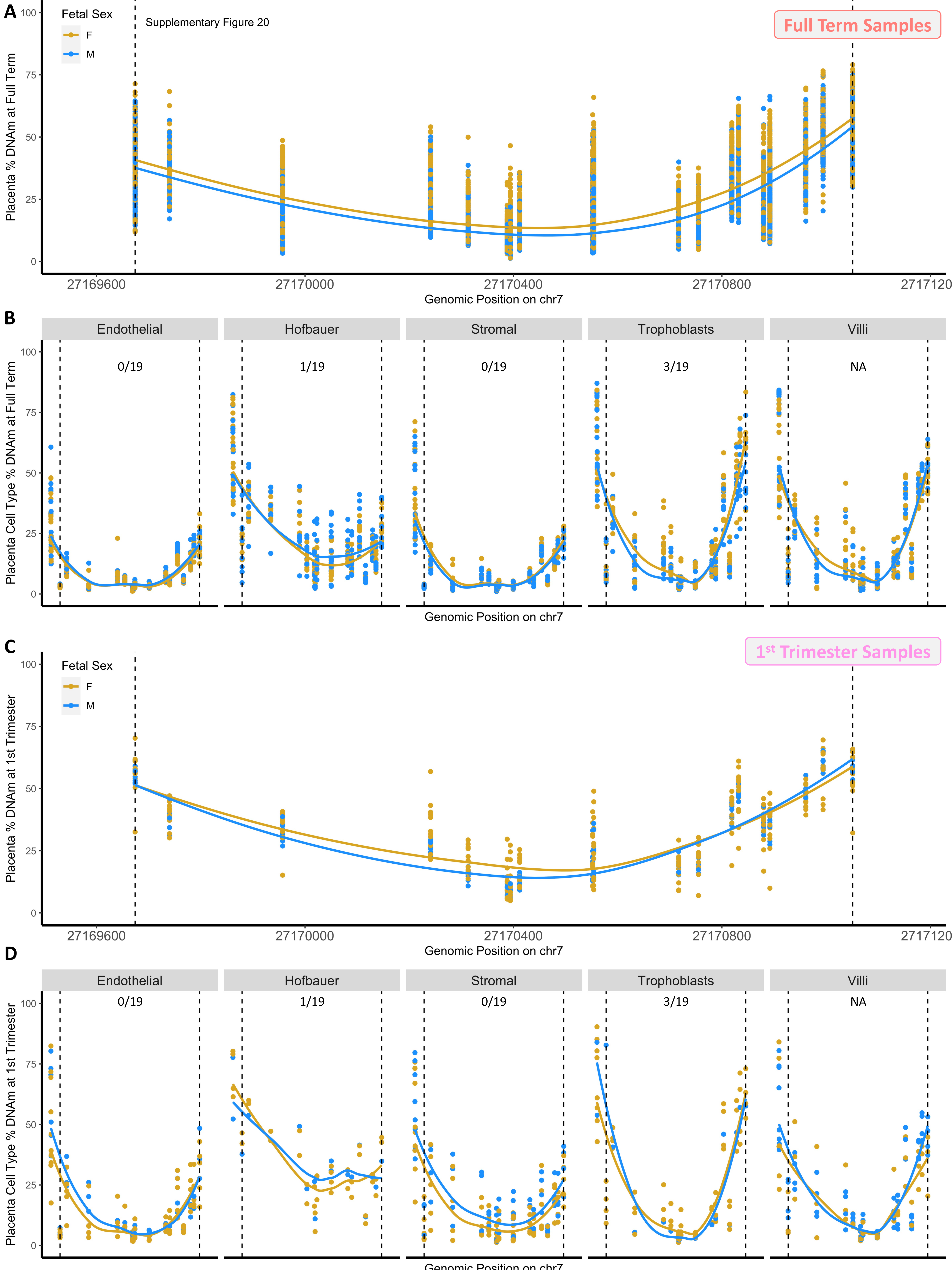
A
Supplementary Figure 20
Full Term Samples
Fetal Sex
F
M
Placenta % DNAm at Full Term
100
75
50
25
0
27169600
27170000
27170400
27170800
2717120
Genomic Position on chr7
B
Endothelial
Hofbauer
Stromal
Trophoblasts
Villi
0/19
1/19
0/19
3/19
NA
Placenta Cell Type % DNAm at Full Term
Genomic Position on chr7
C
1st Trimester Samples
Fetal Sex
F
M
Placenta % DNAm at 1st Trimester
27169600
27170000
27170400
27170800
2717120
Genomic Position on chr7
D
Endothelial
Hofbauer
Stromal
Trophoblasts
Villi
0/19
1/19
0/19
3/19
NA
Placenta Cell Type % DNAm at 1st Trimester
Genomic Position on chr7

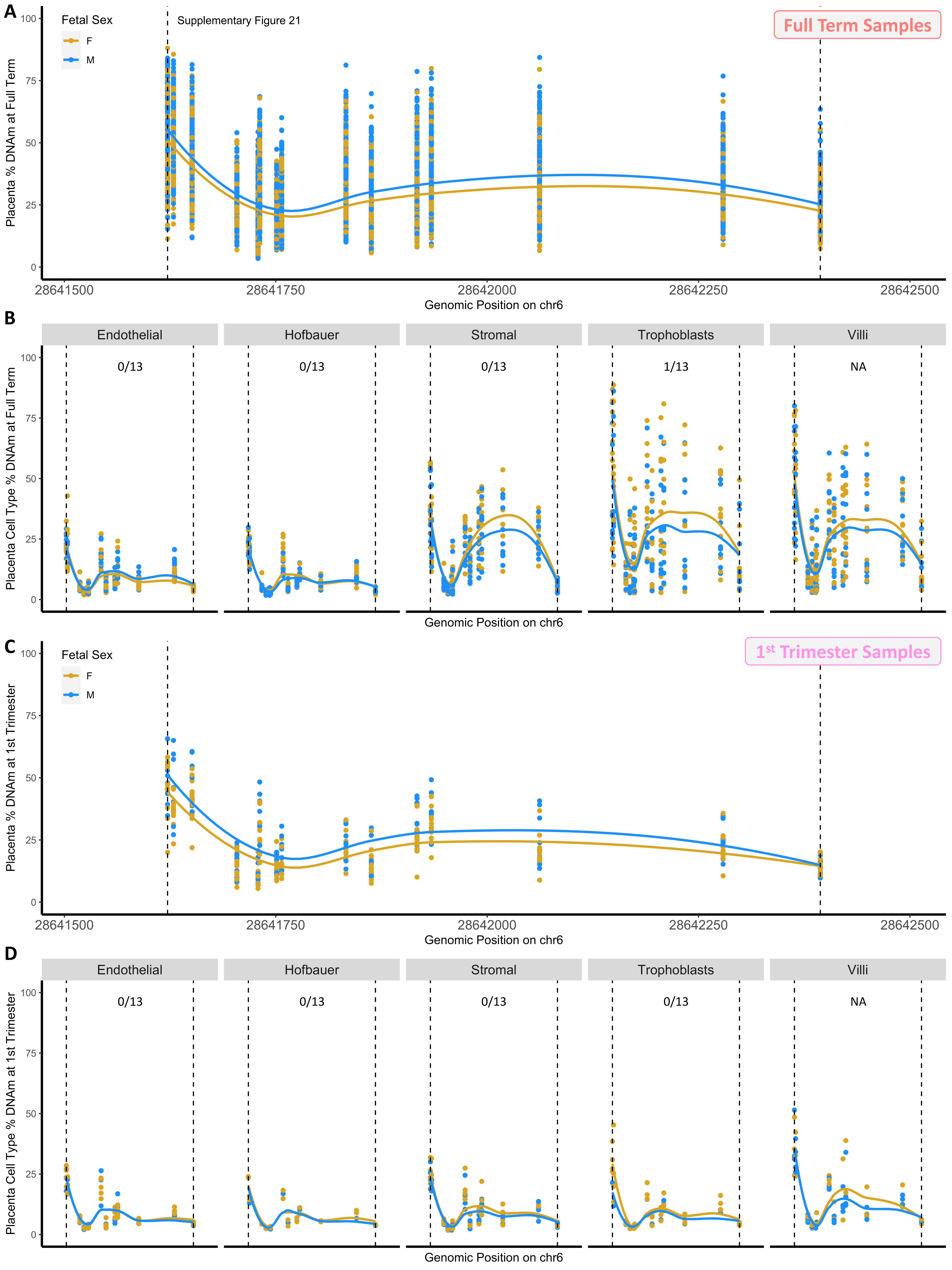
A
Supplementary Figure 21
Full Term Samples
Fetal Sex
F
M
Placenta % DNAm at Full Term
Genomic Position on chr6
B
Endothelial
Hofbauer
Stromal
Trophoblasts
Villi
0/13
0/13
0/13
1/13
NA
Placenta Cell Type % DNAm at Full Term
Genomic Position on chr6
C
1st Trimester Samples
Fetal Sex
F
M
Placenta % DNAm at 1st Trimester
Genomic Position on chr6
D
Endothelial
Hofbauer
Stromal
Trophoblasts
Villi
0/13
0/13
0/13
0/13
NA
Placenta Cell Type % DNAm at 1st Trimester
Genomic Position on chr6
